# Outpatient Follow-Up and 30-Day Readmissions: A Systematic Review and Meta-Analysis

**DOI:** 10.1001/jamanetworkopen.2025.41272

**Published:** 2025-11-04

**Authors:** Ishwarya Balasubramanian, Ellie Bostwick Andres, Chetna Malhotra

**Affiliations:** 1 Lien Centre for Palliative Care, Duke-NUS Medical School, Singapore; 2 Program in Health Services and Systems Research, Duke-NUS Medical School, Singapore

## Abstract

**Importance:**

Outpatient follow-up after discharge has been associated with reduced 30-day readmissions. Since universal follow-up is not feasible, identifying for whom and when outpatient follow-up is most beneficial is essential for optimizing resources and reducing readmissions.

**Objective:**

To quantify the association between outpatient follow-up within 30, 14, and 7 days postdischarge and 30-day all-cause readmissions and assess differences in outcomes by disease, age, and baseline readmission risk.

**Data Sources:**

MEDLINE (via PubMed), Embase, and CINAHL were searched for studies published between January 1, 2000, and August 4, 2025, using terms related to outpatient follow-up and readmissions.

**Study Selection:**

English-language studies assessing the association between outpatient follow-up within 30 days of hospital discharge and 30-day all-cause readmissions among adult inpatients were included.

**Data Extraction and Synthesis:**

Following Preferred Reporting Items for Systematic Review and Meta-Analyses guidelines, 2 reviewers independently screened titles and abstracts. Data were extracted by 1 author and verified by another, and quality assessment was done independently by 2 authors.

**Main Outcomes and Measures:**

The primary outcome was all-cause 30-day readmission. Secondary outcomes included all-cause 30-day emergency department (ED) discharge and mortality. Pooled effect sizes (relative risk ratios [RRRs]) were estimated by disease and age group using multilevel random-effects models.

**Results:**

Eighty-three studies were included in the review and 76 in the meta-analysis. Outpatient follow-up within 30 days vs no follow-up was associated with a reduction in risk of 30-day all-cause readmission (RRR, 0.68; 95% CI, 0.60-0.75), with less reduction (RRR, 0.78; 95% CI, 0.67-0.89) when restricted to studies with low to moderate risk of bias (ROB). Among patients with heart failure (HF) and acute myocardial infarction (AMI), the RRRs for 30-day follow-up in studies with low to moderate ROB were 0.65 (95% CI, 0.48-0.83) and 0.56 (95% CI, 0.32-0.80), respectively. Subgroup analysis using studies with low to moderate ROB showed benefits of 30-day follow-up only among patients aged 65 years or older with HF (RRR, 0.65; 95% CI, 0.48-0.83), AMI (RRR, 0.56; 95% CI, 0.32-0.80), and other diseases such as stroke and chronic obstructive pulmonary disease (RRR, 0.73; 95% CI, 0.59-0.87). Early follow-up vs no follow-up within 14 and 7 days was associated with a significant reduction in readmissions only among patients aged 65 years or older with HF (14 days: RRR, 0.63 [95% CI, 0.40-0.87]; 7 days: RRR, 0.68 [95% CI, 0.47-0.89]) and AMI (14 days: RRR, 0.57 [95% CI, 0.22-0.91]; 7 days: RRR, 0.63 [95% CI, 0.34-0.92]).

**Conclusions and Relevance:**

In this systematic review and meta-analysis, outpatient follow-up within 30 days was associated with reduced 30-day readmissions, but the association varied by patient age and disease type, indicating a need for targeted rather than universal follow-up.

## Key Points

**Question:**

Is outpatient follow-up after hospital discharge associated with reduced risk of readmission among adult patients?

**Findings:**

This systematic review and meta-analysis of 83 studies found outpatient follow-up within 30 days of discharge was associated with reduced risk of 30-day readmission for patients aged 65 years or older, while early follow-up within 7 and 14 days was associated with reduced risk only among patients aged 65 years or older with heart failure or acute myocardial infarction.

**Meaning:**

The findings suggest outpatient follow-up within 30 days of discharge is associated with reduced risk of readmission but follow-up within 7 or 14 days may not be necessary for low-risk patients.

## Teaser

This systematic review and meta-analysis assesses the association between outpatient follow-up within 30 days of discharge and risk of all-cause 30-day readmission among adult patients.

## Introduction

Reducing hospital readmissions is a major policy priority to lower costs and improve care quality and patient outcomes.^1^ National payers,^2^ such as US Medicare under the Hospital Readmissions Reduction Program (HRRP),^3^ penalize hospitals for excessive 30-day readmissions. Outpatient follow-up—already standard practice in many health care systems—is associated with reduced 30-day readmission risk.^4^ However, less is understood about which patients stand to benefit and how soon postdischarge follow-up must occur to reduce readmissions. Given limited health care resources, critically evaluating the complex interactions between patient factors and follow-up timing—a dynamic underexplored in the literature—can offer clinically important and policy-relevant insights.

Findings from existing studies vary regarding follow-up timing, with some suggesting only follow-up within 7 days is associated with reduced 30-day readmission risk^5^ and others finding no benefit from early follow-up.^6^ HRRP penalizes readmissions only among high-risk groups, including those with heart failure (HF), acute myocardial infarction (AMI), chronic obstructive pulmonary disease (COPD), and pneumonia, suggesting interventions to reduce readmissions may not benefit all patients equally.^2^ Randomized clinical trials are the gold standard for causal inference but are often not appropriate or feasible in this context due to ethical concerns of withholding care. Observational studies better reflect diverse clinical and general-population settings but introduce 2 key biases in this context: time-dependent bias, where readmissions before a scheduled follow-up are misclassified as “no follow-up,” thus worsening outcomes for the “no follow-up” group, and confounding bias, where shared factors, such as clinical or social risk, influence the likelihood of both follow-up and readmission (eAppendix 1 in Supplement 1). Inadequate adjustment for biases may undermine reliability of findings. Conflicting findings also arise from variation in populations studied (eg, disease groups), follow-up timing and practitioner types, and outcomes (readmissions only vs composite measures including mortality and/or emergency department [ED] discharges).^7^

In this systematic review and meta-analysis, our primary aim was to synthesize worldwide evidence on the association between outpatient follow-up and 30-day readmissions across disease groups, focusing on when and for whom follow-up is most beneficial, while rigorously evaluating all potential sources of bias. Specifically, we investigated whether the association between outpatient follow-up within 30 days of discharge and all-cause 30-day readmissions varied by disease, age, and other factors. We also investigated whether earlier follow-up (within 7 or 14 days) was associated with lower 30-day readmission risk, and if so, whether this association varied by disease, age, and other risk factors. Our secondary aim was to assess whether associations of outpatient follow-up with 30-day mortality and ED discharges differed from associations with readmissions.

## Methods

This systematic review and meta-analysis followed the Preferred Reporting Items for Systematic Reviews and Meta-Analyses (PRISMA) reporting guideline.^8^ The study is registered with PROSPERO.

### Inclusion and Exclusion Criteria

We included studies of any design examining inpatient hospital admissions among adults discharged home or to the community. The intervention was outpatient follow-up within 30 days (alone or with other postdischarge follow-up components [telephone or home visits]) in any care setting. Studies with outpatient follow-up combined with any predischarge intervention were included only if they reported the numbers who received and did not receive follow-up. The comparator was no known outpatient follow-up within 30 days. The primary outcome was all-cause 30-day readmission. A sensitivity analysis included composite outcomes, combining readmission with ED discharge and/or mortality. Secondary outcomes included all-cause 30-day ED discharge and mortality. We excluded studies not in English, that included psychiatric or obstetric-related admissions or patients younger than 18 years, that evaluated only scheduled follow-up or outcomes beyond or prior to 30 days, and that reported only secondary outcomes.

### Search Strategy and Study Selection

We searched MEDLINE (via PubMed), Embase, and CINAHL for studies published between January 1, 2000, and August 4, 2025, using terms related to outpatient follow-up and readmissions (eTable 1 in Supplement 1). Two reviewers (I.B., E.B.A.) independently screened titles and abstracts using a web and mobile app.^9^ Full texts were retrieved for abstracts included by either reviewer. Both reviewers evaluated full-text articles, resolving conflicts through discussion with the third author (C.M.).

### Data Extraction

We extracted author, publication year, country, population characteristics (number of hospitals or sites, age, disease, sample size [number of admissions], discharge destination, whether patients had multiple admissions, time period, and exclusions), intervention details (time to follow-up, practitioner type [eg, primary care practitioner]), additional intervention components, data source for outpatient follow-up (eg, medical records), comparator description, and outcome description (eg, readmission, mortality, or composite outcome). For the meta-analysis, we extracted the number of patients with and without follow-up, number of readmissions, and effect sizes (odds ratios [ORs] or hazard ratios [HRs] and associated 95% CIs for each outcome) and analysis details (statistical methods, adjustments for bias, and handling of missing data).

### Risk of Bias Assessment

We used Risk of Bias in Non-Randomized Studies of Interventions (ROBINS-I)^10^ to assess risk of bias (ROB) across 7 domains (eAppendix 1 in Supplement 1). Overall ROB was categorized as low (all domains low), moderate (all domains low or moderate), serious (at least 1 domain serious, none critical), or critical (at least 1 domain critical).

### Statistical Analysis

We conducted a meta-analysis to estimate the association of outpatient follow-up with 30-day all-cause readmission, using adjusted effect sizes when available. HRs were treated as equivalent to relative risk ratios (RRRs), given rare events and short follow-up time.^11^ ORs were converted to RRRs using Zhang and Yu’s^12^ formula. When relevant parameters were unavailable, ORs and RRRs were assumed equal, which is true when outcome rates are low,^13^ as they were in most studies. When neither HRs nor ORs were reported, we calculated crude risk ratios (CRRs) using reported data (eAppendix 2 in Supplement 1). We included all estimates from studies reporting results stratified by disease or follow-up timing. For studies with data stratified by other categories (eg, practitioner type), we combined estimates using fixed effects.

We estimated pooled effect size (and 95% CI) for the association of 30-day follow-up with 30-day all-cause readmission by disease and age group for all studies with low to moderate ROB separately, using multilevel random-effects models to account for between-study correlation. Further subgroup analyses evaluated whether early follow-up (within 7 or 14 days) was associated with reduced 30-day readmissions for diseases or age groups that benefited from 30-day follow-up. *I*^2^ was used to assess heterogeneity. Since *I*^2^ was expected to be high given variability in study populations, follow-up timing, practitioners, and methods used for bias control, we conducted a meta-regression with sample readmission risk (eAppendix 3 in Supplement 1) as a covariate to further explore sources of heterogeneity.

Sensitivity analyses included pooling composite outcomes when available, a leave-1-out analysis to check for influence of outliers, and a meta-regression using effect size type (HR, RRR, OR, or CRR) as a covariate. A meta-regression assessed the association of each ROB domain and major biases with the overall effect size. Publication bias was assessed using the Egger test; where present, adjusted effect sizes using the Duval and Tweedie trim-and-fill method were reported. Stata, version 18.0 (StataCorp LLC), was used for all analyses. Two-sided *P* < .05 was considered significant. Results for secondary outcomes and other moderators (readmission risk factors and practitioner type) for which meta-analysis was not possible are presented through narrative synthesis.

## Results

### Study Selection and Characteristics

**Figure 1.**
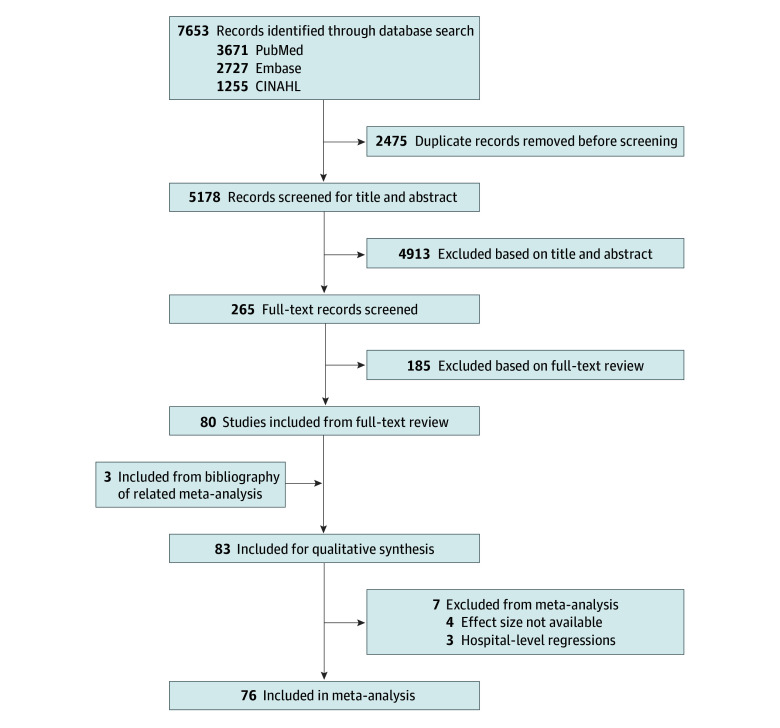
Study Selection Flowchart

The initial search identified 7653 studies. After removing duplicates, 5178 titles and abstracts were screened, 265 underwent full-text review, and 80 were included. Three additional studies were identified from related reviews,^14,15^ bringing the total number of studies to 83 for narrative synthesis.^5,6,16,17,18,19,20,21,22,23,24,25,26,27,28,29,30,31,32,33,34,35,36,37,38,39,40,41,42,43,44,45,46,47,48,49,50,51,52,53,54,55,56,57,58,59,60,61,62,63,64,65,66,67,68,69,70,71,72,73,74,75,76,77,78,79,80,81,82,83,84,85,86,87,88,89,90,91,92,93,94,95,96^ (Figure 1).

Studies were published between 2010 and 2025 (eTable 2 in Supplement 1), with all but 14 ([16.9%]^16,19,23,25,26,30,37,40,52,62,78,80,88,95^) from the US. Sample sizes ranged from 65^69^ to 749 402,^18^ including general inpatients or disease-specific admissions. Four studies (4.8%)^16,17,18,19^ evaluated multiple disease groups and 9 (10.8%)^16,20,29,30,59,70,77,85,87^ assessed multiple follow-up time intervals, resulting in 109 total assessments, including admissions for general inpatients (33 [30.3%]) and inpatients with HF (22 [20.2%]), COPD (9 [8.3%]), postoperative status (11 [10.1%]), AMI (8 [7.3%]), stroke (5 [4.6%]), diabetes (3 [2.8%]), pneumonia (2 [1.8%]), atrial fibrillation (2 [1.8%]), irritable bowel disease (2 [1.8%]), outpatient parenteral therapy (2 [1.8%]), HIV (2 [1.8%]), lupus (2 [1.8%]), trauma (2 [1.8%]), cirrhosis (1 [0.9%]), sickle cell (1 [0.9%]), sepsis (1 [0.9%]) and seizure or epilepsy (1 [0.9%]). Over half of the studies (44 [53.0%])^6,16,18,19,20,22,23,24,25,26,27,28,29,30,35,36,38,39,40,41,48,49,54,58,59,65,68,70,73,74,75,76,78,80,81,82,83,84,85,86,87,88,91,93^ evaluated follow-up within 14 days, of which 27 (61.4%)^6,16,18,19,20,22,24,25,26,27,29,30,35,38,40,48,58,68,70,73,78,81,83,85,86,87,93^ assessed follow-up within 7 days.

Of the 83 studies included, 7 (8.4%) were excluded from meta-analysis for reporting only hospital-level regressions^20,38,39^ or insufficient statistics for effect size calculations^47,70,75,86^; the other 76 studies (91.6%) were included in the meta-analysis. Three studies (3.6%) reported only composite outcomes^30,37,78^ and were included only in sensitivity analyses. The meta-analysis of all-cause readmissions included all but these 10 studies (12.0%)^20,30,37,38,39,47,70,75,78,86^ (92 assessments [84.4%]).

### Readmission and Outpatient Follow-Up Rates

All-cause readmission rates varied widely: 3.7% to 30.8% for general inpatients, 13.6% to 31.9% for HF, 9.0% to 19.4% for COPD, 6.9% to 23.0% for AMI, and 7.3% to 13.7% for stroke. Seven-day outpatient follow-up rates also varied, ranging from 8.5% to 74.9% for HF and 24.1% to 61.5% for general inpatients (eTable 2 in Supplement 1).

#### ROB Assessment

Only 1 study had a low ROB.^20^ Most studies had critical (30 [36.1%]),^22,25,34,35,36,40,41,42,51,55,56,60,61,62,64,65,66,67,69,72,73,76,77,78,80,82,86,87,88,91^ serious (26 [31.3%]),^21,24,28,29,32,33,44,45,46,47,48,49,50,52,53,63,68,70,71,74,75,81,84,90,92,94^ or moderate (26 [31.3%])^5,6,16,17,18,19,23,26,27,30,31,37,38,39,43,54,57,58,59,79,83,85,89,93,95,96^ ROB. Of note, critical and serious ROB ratings were often for the outcome measurement domain (47 of 78 studies [60.3%]^21,22,25,28,29,32,33,34,35,36,41,42,45,46,49,50,51,52,53,55,56,60,61,62,63,64,65,66,67,69,72,73,74,76,77,78,80,81,82,84,86,87,88,90,91,92,94^) (eFigure and eTable 3 in Supplement 1).

#### Meta-Analysis

Outpatient follow-up within 30 days vs no follow-up was associated with a reduced risk of 30-day all-cause readmission (RRR, 0.68; 95% CI, 0.60-0.75). There was less reduction in associated risk (RRR, 0.78; 95% CI, 0.67-0.89) when restricted to low to moderate ROB studies (34 assessments [31.2%]).

### Subgroup Analysis by Moderators of Disease, Follow-Up Time, Age, and Sample Risk Rating

For HF (16 assessments [14.7%]) and AMI (6 assessments [5.5%]), 30-day outpatient follow-up vs no follow-up was associated with reduction in readmission risk (HF: RRR, 0.66 [95% CI, 0.55-0.78]; AMI: RRR, 0.64 [95% CI, 0.37-0.91]), consistent when restricted to studies with low to moderate ROB (HF [8 assessments (9.6%)]: RRR, 0.65 [95% CI, 0.48-0.83]; AMI [5 (6.0%)]: RRR, 0.56 [95% CI, 0.32-0.80]). For stroke (5 assessments [6.0%]), pneumonia (2 [2.4%]), and postoperative status (11 [13.3%]), 30-day outpatient follow-up was associated with reduced readmission risk (RRRs of 0.67 [95% CI, 0.41-0.92] for stroke, 0.57 [95% CI, 0.53-0.61] for pneumonia, and 0.55 [95% CI, 0.35-0.75] for postoperative care), but there were insufficient studies with low to moderate ROB for separate analysis. For COPD (7 assessments [8.4%]), there was no association overall (RRR, 0.83; 95% CI, 0.64-1.02) or for studies with low to moderate ROB (3 assessments [3.6%]) (RRR, 0.76; 95% CI, 0.43-1.08). Outpatient follow-up was associated with reduced readmission risk among general inpatients (26 assessments [31.3%]) (RRR, 0.68; 95% CI, 0.56-0.80), though the effect attenuated considerably when restricted to evidence from studies with low to moderate ROB (RRR, 0.88; 95% CI, 0.80-0.96) (Table).

**Table.** Association of 30-Day Outpatient Follow-Up With All-Cause 30-Day Readmissions Overall and by Disease

Study population	All studies				Studies with low to moderate ROB			
	No. (%)		Pooled RRR (95% CI)	*I* ^2^	No. (%)		Pooled RRR (95% CI)	*I* ^2^
	Studies	Assessments			Studies (n = 73)	Assessments (n = 92)		
All studies	73 (100)	92 (100)	0.68 (0.60-0.75)	99.81	21 (29)	34 (37)	0.78 (0.67-0.89)	99.91
Heart failure	14 (19)	16 (17)	0.66 (0.55-0.78)	99.52	7 (10)	8 (9)	0.65 (0.48-0.83)	99.64
COPD	7 (10)	7 (8)	0.83 (0.64-1.02)	98.74	3 (4)	3 (3)	0.76 (0.43-1.08)	99.74
Stroke	5 (7)	5 (5)	0.67 (0.41-0.92)	94.84	1 (1)	1 (1)	0.98 (0.97-0.99)	NA
AMI	5 (7)	6 (7)	0.64 (0.37-0.91)	99.78	4 (5)	5 (5)	0.56 (0.32-0.80)	99.84
Postoperative	8 (11)	11 (12)	0.55 (0.35-0.75)	97.15	2 (3)	2 (2)	0.62 (0.06-1.18)	98.84
Pneumonia	2 (3)	2 (2)	0.57 (0.53-0.61)	82.03	1 (1)	1 (1)	0.55 (0.54-0.57)	NA
General inpatients	23 (32)	26 (28)	0.68 (0.56-0.80)	99.81	7 (10)	9 (10)	0.88 (0.80-0.96)	99.57

Abbreviations: AMI, acute myocardial infarction; COPD, chronic obstructive pulmonary disease; NA, not available; ROB, risk of bias; RRR, relative risk ratio.

**Figure 2.**
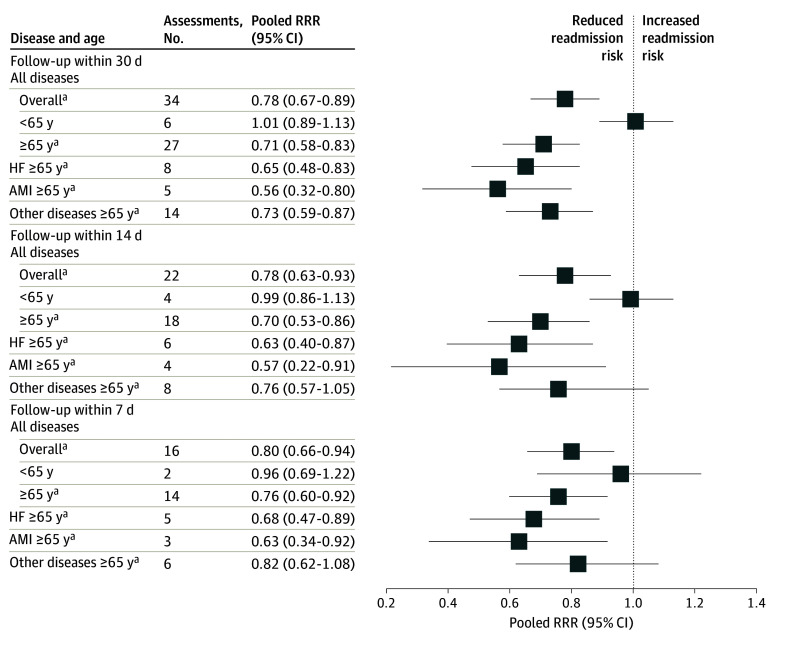
Association of 30-Day Outpatient Follow-Up With All-Cause 30-Day Readmissions by Disease and Age (Studies With Low to Moderate Risk of Bias Only) Age cutoffs indicate that the median or mean age of the sample was younger than 65 years or 65 years or older. There were no studies of patients with heart failure (HF) or acute myocardial infarction (AMI) with mean or median age less than 65 years. Other diseases included chronic obstructive pulmonary disease, stroke, diabetes, pneumonia, atrial fibrillation, irritable bowel disease, outpatient parenteral therapy, HIV, lupus, trauma, cirrhosis, sickle cell disease, sepsis, and epilepsy as well as general inpatients and postoperative patients. RRR indicates relative risk ratio. ^a^*P* < .05.

Subgroup analysis by age and disease group showed 30-day outpatient follow-up vs no follow-up was associated with reduced readmission risk for all age and disease groups (eTable 4 in Supplement 1). However, when restricted to studies with low to moderate ROB (Figure 2), 30-day outpatient follow-up was only associated with reduced readmission risk for patients aged 65 years or older (overall [ie, all diseases]: RRR, 0.71 [95% CI, 0.58-0.83]; HF: RRR, 0.65 [95% CI, 0.48-0.83]; AMI: RRR, 0.56 [95% CI, 0.32-0.80]; other diseases: RRR, 0.73 [95% CI, 0.59-0.87]). Early follow-up within 14 and 7 days vs no follow-up was associated with reduced readmissions only among patients aged 65 years or older with HF or AMI (HF within 14 days: RRR, 0.63 [95% CI, 0.40-0.87]; within 7 days: RRR, 0.68 [95% CI, 0.47-0.89]; AMI within 14 days: RRR, 0.57 [95% CI, 0.22-0.91]; within 7 days: RRR, 0.63 [95% CI, 0.34-0.92]).

*I*^2^ remained high in subgroup analyses. In meta-regression using sample risk rating as a covariate (eTable 5 in Supplement 1), the effect of 30-day follow-up varied by risk categories within subgroups. Outpatient follow-up was associated with reduced readmission risk in low- and medium-risk patients aged 65 years or older and high-risk patients aged younger than 65 years.

### Sensitivity Analyses

Results from the sensitivity analysis with composite outcomes (eTable 6 in Supplement 1) affirm the main results. There was no publication bias in the subgroup analysis. Trim-and-fill analysis for overall effect of 30-day outpatient follow-up yielded results similar to the main findings (RRR, 0.68; 95% CI, 0.61-0.75). Meta-regression showed no significant difference in effect sizes related to the type of statistic used but varied by ROB, being significantly lower in studies with critical ROB (eTable 7 in Supplement 1). In particular, studies with bias in the outcome domain, especially time-dependent bias, reported lower RRR (eTable 8 in Supplement 1). Leave-1-out analysis confirmed none of the studies unduly attenuated the overall results.

### Narrative Synthesis

#### Other Factors Associated With Readmission

Several studies suggested a differential effect of outpatient follow-up based on predicted readmission risk. Nguyen et al^83^ found follow-up was associated with reduced readmissions only among high-risk medicine patients (LACE [length of stay, acuity of admission, comorbidities, and recent ED use] index ≥11), not low-risk or postoperative patients. Tong et al^79^ reported smaller reductions in readmission rate as predicted readmission risk increased and no significant reduction for very high-risk patients. Similarly, an HF study found significant associations with reduced readmissions for patients with 1 comorbidity or a length of stay (LOS) of 7 to 13 days but not for patients with no or multiple comorbidities or LOS less than 7 or more than 13 days.^30^ These findings support our meta-regression results: follow-up was not associated with reduced readmissions among patients with very low or very high readmission risk. Jackson et al^70^ found no association between 7-day follow-up and readmissions for low-risk patients, suggesting optimal follow-up timing may vary by patient readmission risk.

#### Type of Clinician

Six studies (7.2%) evaluated the association between specialist follow-up and readmissions, finding either no association or increased all-cause 30-day readmissions.^16,20,21,37,44,85^ One study of general inpatients (1.2%)^85^ found specialist follow-up within 7 and 14 days was associated with increased all-cause 30-day readmissions, while follow-up within 30 days was associated with reduced readmissions.^85^ Findings were similar for patients with HF^16,20^ and stroke.^44^ Three studies (3.6%) evaluated outpatient follow-up with nonphysician practitioners^17,41,85^ and 6 (7.2%) in outpatient clinics staffed by physicians and nonphysicians,^35,64,66,73,82,91^ all showing significant associations with reduced readmissions.

#### Secondary Outcomes

Fifteen studies (18.1%) evaluated mortality as an outcome, either with readmissions (6 [7.2%])^23,29,30,32,37,38^ or separately (9 [10.8%]).^19,20,25,32,38,58,59,75,84^ Two (2.4%) found 30-day outpatient follow-up was associated with reduced readmissions but not mortality,^20,25^ 2 (2.4%) found a protective association for both outcomes,^75,84^ and 2 (2.4%) found no association for either.^19,38^ Three studies (3.6%) found outpatient follow-up was associated with increased readmissions but lower mortality.^32,58,59^

Similarly, 10 studies (12.0%) evaluated the association between follow-up and both readmissions and ED discharges,^31,32,45,54,56,59,67,75,84,88^ with 5 (6.0%) showing no association for either outcome,^32,56,59,67,88^ 3 (3.6%) showing associations with reduced ED discharges and readmissions,^31,45,75^ 1 (1.2%) showing no association with readmissions but associations with reduced ED discharges,^54^ and 1 (1.2%) showing an association with reduced readmissions but not ED discharges.^84^

## Discussion

This systematic review and meta-analysis advances current understanding of the association between outpatient follow-up and 30-day readmission risk considering study ROB, patient age, disease type, and timing of follow-up. A recent meta-analysis, limited to US studies and 4 disease groups, showed reduced risk associated with outpatient follow-up among patients with HF and stroke.^14^ An international review also reported reduced risk of readmission associated with early physician follow-up but did not distinguish patient subgroups, follow-up intervals, and visit types (including home visits), limiting its applicability for prioritizing follow-up.^15^ We found 30-day follow-up was associated with a 32% lower readmission risk overall and 22% lower risk in studies with low to moderate ROB, suggesting studies that fail to adjust for bias may overstate benefit.

Our findings also showed that the association between follow-up and readmissions varied, with greater benefit for patients aged 65 years or older and populations with HF and AMI. Evidence from studies with low to moderate ROB showed follow-up was associated with reduced readmissions by 35% among patients with HF and 44% in patients with AMI but no significant reduction in patients with COPD, substantiating previous reviews.^14,97^ Likewise, evidence from studies with low to moderate ROB showed only a slight reduction in readmissions for patients with stroke and no associations for postoperative and general inpatients.^6,79,83,89^ This differential outcome may reflect disease-specific considerations impacting the value of follow-up in reducing readmissions. For example, while outpatient follow-up facilitates adjustment of treatment plans, medication adherence,^98^ and early detection of complications, potentially reducing readmissions among patients with HF or AMI, it may provide less value for patients with COPD, whose readmissions are often driven by comorbidities.^99,100^ In this study, among adults aged 65 years or older, 30-day follow-up was associated with a 29% reduction in readmissions irrespective of disease, which could reflect their increased readmission risk due to comorbidities, frailty, cognitive impairment, and functional limitations.^101^

The association between early follow-up (within 7 or 14 days) and readmissions also differed across patient groups, with benefits for readmission risk only apparent among adults aged 65 years or older with HF or AMI. Despite broad endorsement of 7- and 14-day follow-up as quality metrics,^102^ we found no evidence supporting universal recommendations for early follow-up rather than 30-day follow-up. Disease-specific recommendations, such as the American Heart Association’s Get With the Guidelines endorsement of 7-day follow-up for patients with HF, are more aligned with evidence.^103^ We found that among patients aged 65 years or older with conditions other than HF or AMI, 30-day follow-up was associated with reduced readmissions, but earlier follow-up may confer no additional advantage. Meanwhile, no evidence from studies with low to moderate ROB supported recommendations for 30-day follow-up for patients younger than 65 years with conditions other than HF or AMI. Meta-regression and narrative synthesis results highlighted additional readmission risk factors—comorbidities and LOS—to consider when assessing follow-up benefit. Validated risk prediction tools—such as the probability of repeated admission,^104^ LACE index,^105^ or Community Assessment Risk Screen^106^—may allow for more nuanced triage strategies. There is also limited evidence favoring specific practitioner types. More robust studies are needed to clarify the relationship between readmissions and follow-up with nonphysician practitioners.

Our review highlights a major ROB gap in existing studies, as indicated by the small proportion of low-moderate ROB studies and the significant variation in estimates of reduced readmission risk between all studies and studies with low to moderate ROB. Studies with low to moderate ROB consistently showed smaller reductions in readmission risk, indicating studies with higher ROB reported more favorable findings than studies with low-moderate ROB. In particular, studies that did not adjust for time-dependent bias may have overstated the association between outpatient follow-up and readmissions, and the true effect is likely smaller than that reported by studies with higher ROB. This has implications for hospital systems when setting realistic readmission targets. We have synthesized practical guidance to improve study quality and rigor going forward (Box). To enhance the utility of future research, we especially stress the use of clear definitions of interventions and outcomes to assess bias and guide future implementation and careful use of appropriate statistical methods to control for bias, such as inverse probability weights to control for confounding and time-varying models to control for time-dependent bias. All studies assessing readmission risk should also assess mortality, as the 2 are competing risks.^107^

Box. **Recommendations for Best Practices in Academic Research Concerning the Association of Outpatient Follow-Up With Outcomes**

**Population**

- Select a representative sample of patients, ideally from multiple hospitals.
- Describe the patient risk profile. This can be done using indicators such as age, primary disease, comorbidities, risk prediction score, and social risk factors.

**Intervention**

- Clearly define the outpatient follow-up, specifying the practitioner and components of follow-up.
- Describe the prevalence of outpatient follow-up across different time intervals (eg, 0-7 days, 0-14 days, or 0-30 days) and the proportion of patients in each time interval who received follow-up, did not receive follow-up, or were readmitted or died before follow-up.
- Describe the risk profiles of the patients in the outpatient follow-up and no follow-up categories.

**Outcome**

- Account for competing risk of mortality along with readmissions or use a composite outcome of readmissions and mortality.
- Focus only on unplanned readmissions.

**Analysis**

- Control for confounding using appropriate statistical methods such as inverse probability weights, case-control design, and instrumental variables.
- Use a time-variable model to control for time-dependent bias.
- If mortality is not part of the composite outcome, treat it as a competing risk.
- Address violations of the proportionality assumption in time-to-event analysis.

### Strengths and Limitations

Our review’s strengths are its comprehensive scope and rigorous quality evaluation, ensuring robust findings. However, there are limitations. First, heterogeneity is a major concern given differences in populations, intervention components, follow-up timing, and bias-control methods and persisted in our subgroup analysis. However, such heterogeneity is policy-relevant, highlighting our central message that outpatient follow-up is associated with reduced readmissions but the association varies across patient groups. Second, most studies were US-based, limiting generalizability and highlighting the need for more research in diverse health systems. Third, subgroup analyses were limited by few studies and the potential for misclassification (such as general inpatients misclassified as not having HF or AMI) or incorrect age group classification based on sample mean or median. Such misclassification likely underestimated follow-up benefits for high-risk patients aged 65 years or older. Fourth, consistent with prior reviews,^108,109^ we combined various effect measures (HR, OR, RRR, and CRR) in our meta-analysis as justified by short follow-up time, rare-event assumption, and meta-regression showing no significant differences in effect size. Fifth, despite extensive efforts, some relevant studies could have been missed.

## Conclusions

In conclusion, our systematic review and meta-analysis offers compelling evidence on the association between outpatient follow-up and reduced readmissions. Rather than universal recommendations, risk factors such as patient age and disease should be considered in prioritizing postdischarge follow-up. We emphasize the need for high-quality studies and offer actionable recommendations to guide future research.

## Notes

Supplement 1. **eAppendix 1.** Main Biases and Domains in the Quality Assessment of Studies **eTable 1.** Search Strategy **eAppendix 2.** Formulas Used for Effect Sizes **eAppendix 3.** Assessing Readmission Risk of Sample in Good-Quality Studies **eTable 2.** Summary of Included Studies **eTable 3.** Quality Assessment of Studies: ROBINS–I **eFigure.** Summary of Bias Across Studies **eTable 4.** Meta-Analysis Results—Subgroup Analysis by Disease, Age, and Time to Outpatient Follow-Up **eTable 5.** Meta-Regression Results Using Risk Score as a Predictor **eTable 6.** Sensitivity Analysis Results: Meta-Analysis Using Composite Outcome When Available **eTable 7.** Meta-Regression Results Evaluating the Effect of Statistic Used and Quality of Studies **eTable 8.** Meta-Regression Results Evaluating the Effect of Quality Domains and Specific Biases
eReferences

Supplement 2.
Data Sharing Statement
