# Supplementary material for: Outpatient Follow-Up and 30-Day Readmissions: A Systematic Review and Meta-Analysis
Source: JAMA Netw Open. 2025 Nov 4;8(11):e2541272. doi: 10.1001/jamanetworkopen.2025.41272 (PMC12587199; doi:10.1001/jamanetworkopen.2025.41272)
Supplement: Supplement 1. — eAppendix 1. Main Biases and Domains in the Quality Assessment of Studies eTable 1. Search Strategy eAppendix 2. Formulas Used for Effect Sizes eAppendix 3. Assessing Readmission Risk of Sample in Good-Quality Studies eTable 2. Summary of Included Studies eTable 3. Quality Assessment of Studies: ROBINS–I eFigure. Summary of Bias Across Studies eTable 4. Meta-Analysis Results—Subgroup Analysis by Disease, Age, and Time to Outpatient Follow-Up eTable 5. Meta-Regression Results Using Risk Score as a Predictor eTable 6. Sensitivity Analysis Results: Meta-Analysis Using Composite Outcome When Available eTable 7. Meta-Regression Results Evaluating the Effect of Statistic Used and Quality of Studies eTable 8. Meta-Regression Results Evaluating the Effect of Quality Domains and Specific Biases eReferences [file jamanetwopen-e2541272-s001.pdf]

Supplementary Online Content

Balasubramanian I, Andres EB, Malhotra C. Outpatient follow-up and 30-day readmissions: a systematic review and meta-analysis. *JAMA Netw Open*. 2025;8(11):e2541272. doi:10.1001/jamanetworkopen.2025.41272

eAppendix 1. Main Biases and Domains in the Quality Assessment of Studies
eTable 1. Search Strategy
eAppendix 2. Formulas Used for Effect Sizes
eAppendix 3. Assessing Readmission Risk of Sample in Good-Quality Studies
eTable 2. Summary of Included Studies
eTable 3. Quality Assessment of Studies: ROBINS–I
eFigure 1. Summary of Bias Across Studies
eTable 4. Meta-Analysis Results—Subgroup Analysis by Disease, Age, and Time to Outpatient Follow-Up
eTable 5. Meta-Regression Results Using Readmission Risk Score as a Predictor
eTable 6. Sensitivity Analysis Results: Meta-Analysis Using Composite Outcome When Available
eTable 7. Meta-Regression Results Evaluating the Effect of Statistic Used and Quality of Studies
eTable 8. Meta-Regression Results Evaluating the Effect of Quality Domains and Specific Biases
eReferences

This supplementary material has been provided by the authors to give readers additional information about their work.

© 2025 Balasubramanian I et al. *JAMA Network Open*.

eAppendix 1: Main biases and domains in the quality assessment of studies

The quality of included studies was assessed by **ROBINS-I** which evaluates bias across seven domains. Below, we describe biases under each domain in the specific context of this review.

1.Bias due to confounding occurs when a external factor—known as a confounder—influences both the intervention (in this case, receiving outpatient follow-up) and the outcome (readmission), which may lead to overestimation or underestimation of the true association of intervention and outcome. For example, if patients who receive outpatient follow-up are more likely to have a higher baseline risk (e.g. age, comorbidities) and those same factors increase the risk of readmissions, then any observed association between follow-up and readmissions may be driven by these underlying differences, rather than the follow-up itself.

In our context, we identified the following domains that may potentially confound the relationship between outpatient follow-up and readmissions – *patient demographic characteristics* (eg., age, sex, race, socio-economic characteristics), *patient clinical characteristics* (co-morbidities, risk score), *index admission characteristics* (length of stay) and *hospital level characteristics* (quality indicators, no of beds). We assessed studies as having low Risk of Bias (RoB) in this domain if they used statistical methods to control for confounding (eg., propensity score matching, target randomized trial emulation, instrumental variables) or controlled confounding by design (eg., case-control, hospital level regression). If at least one variable in each of the above domains were included as confounders in the regression model, we rated studies as having moderate RoB. If there were no confounders to represent one or more of the above domains, studies were rated as having serious RoB. If univariate models or proportions are presented, studies were rated as having critical RoB in this domain.

2. Bias due to measurement of outcomes occurs when the outcome is inaccurately assessed. In our context, two major concerns are *mortality outcome bias* and *time-dependent bias,* both of which can seriously affect validity of results.

Time-dependent bias occurs because both outpatient follow-up and readmissions are dynamic. Readmission or death are competing risks for follow-up – once a patient is readmitted or dies, they can no longer receive an outpatient follow-up within the time frame. Many studies, however, define both follow-up and readmissions as binary variables, leading to four mutually exclusive categories (A, B, C and D in the figure below). This simplification introduces misclassification: for instance, patients who are readmitted before having a follow-up are misclassified in category C (no follow-up and readmitted group), artificially worsening outcomes for the 'no follow-up group'. This misclassification exemplifies time-dependent bias- a bias introduced by ignoring the timing and sequence of events. Studies that modelled follow-up as a time-varying covariate were therefore assessed as having no time-dependent bias.

Mortality outcome bias arises because death is a competing risk for readmission. Patients who die within 30 days cannot be readmitted, but if they are still counted when defining readmission as the outcome, misclassifies these patients in category B or D. To mitigate this, studies should either (i) use a composite outcome (including readmissions and mortality) (ii) apply a cause-specific hazard model (censoring at death), or (iii) use other statistical model to account for mortality as a competing risk (e.g., Fine-Gray sub-distribution hazard models). For quality assessment, studies employing any of these approaches were assessed as having no mortality outcome bias.

Finally, ***misclassification bias*** may also occur when readmissions are measured only within a single hospital's data system. Patients readmitted elsewhere would be missed, underestimating the true outcome rate.

3. Bias in selection of participants occurs when there are systematic differences between those who are included and those not included in the study. In this context, two common sources are:

Immortal time bias occurs when patients who die or are readmitted before outpatient follow-up are excluded, in an attempt to avoid time dependent bias. This creates an "immortal time" during which

© 2025 Balasubramanian I et al. *JAMA Network Open*.

patients must remain alive and event-free to qualify for inclusion, thereby artificially inflating survival or time without readmission.

Mortality exclusion bias occurs when studies exclude patients who die within 30 days to avoid mortality outcome bias.

These exclusions result in a selected sample that is systematically healthier than the full patient population.

In addition to these, selection bias may also arise when there are other *systematic exclusions* (e.g., exclusion of high-risk patients) or if *multiple admissions per patient are included* without accounting statistically for the within-patient correlation, which can overweight certain individuals in the analysis.

4. Bias in classification of interventions occurs when there is a misclassification of the intervention, i.e. misclassifying whether a patient received follow-up or not. This bias can arise if follow-up status is not identified from administrative records (for e.g., reported by nurse or self-report). It can also arise if the intervention is not clearly defined (for e.g., follow-up provider or follow-up time is not clearly mentioned). This bias can also arise if outpatient follow-up is defined as receiving follow-up *before the first readmission* as we are interested in the effect of follow-up on readmissions. Classifying follow-up at any time in the 30 days, even after readmission can be a critical bias.

5. Bias due to deviations from intended interventions occurs when there are differences between what was intended and what was received. This can happen if there is low adherence to the intervention. Since we included studies only with actual outpatient follow-ups and not scheduled outpatient follow-ups, we do not expect this bias. However, this bias can arise if the non-intervention group inadvertently receives follow-up for e.g., if outpatient follow-up is defined as follow-up in a specific hospital or specialized clinic (in which case, the no follow-up group may not have received outpatient follow-up in the specific hospital but may have followed up elsewhere).

6. Bias due to missing data occurs when there is missing data on outcomes, intervention or covariates and if this missing data is likely not random.

7. Bias in selection of reported result if there is *selective outcome reporting* (in our context, if there are multiple outcomes e.g. all-cause readmission, specific-cause readmission, mortality etc., but only significant or favorable results are reported), *selective analysis reporting* (for e.g., reporting only unadjusted results and omitting adjusted results since they are not favorable) or *selective sub-group reporting* (for e.g., reporting results for high-risk patients only and not for the entire sample)

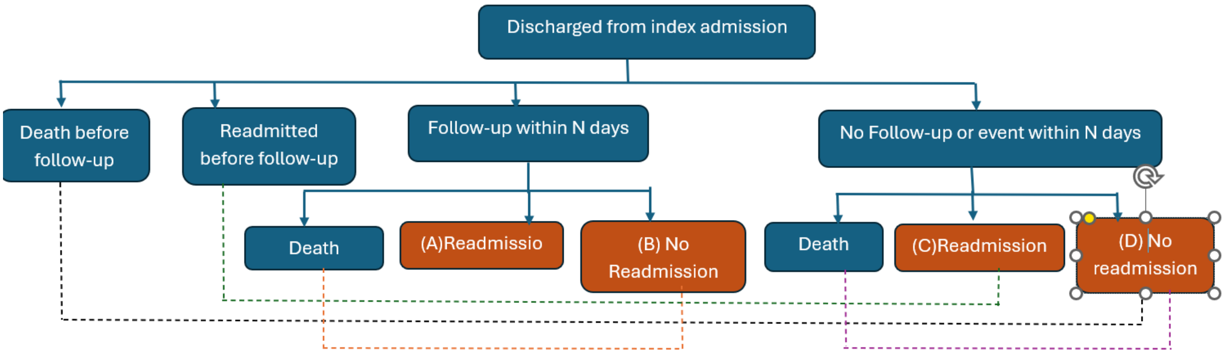

© 2025 Balasubramanian I et al. *JAMA Network Open*.

We used the following criteria to objectively assess the risk of bias.

Risk of Bias rating	Bias due to confounding	Bias in selection of participants	Bias in classification of interventions	Bias due to deviations from intended interventions	Bias due to missing data	Bias in measurement of outcomes	Bias in selection of the reported result
Critical	Univariate analysis/proportions are reported	Systematic exclusion (including Immortality Time Bias) AND Mortality Exclusion Bias AND Multiple admissions per patient are used with no model to control for within-patient correlation	Follow-up status is not identified from administrative records AND Follow-up status is defined as occurring before/after readmission AND No pre-specified definition of outpatient follow-up	Not applicable	>20% excluded observations from the initial sample to analytical sample due to missing data	Time variable bias AND Mortality outcome bias AND Misclassification of outcome	Selective outcome AND Selective analysis AND Selective sub-group reporting
Serious	Some confounding domains are included in statistical models	Any two of the above	Any two of the above	If follow-up is restricted to one specific hospital	10-20%	Any two of the above	Any two of the above
Moderate	All confounding domains are included in statistical models	Any one of the above	Any one of the above	If follow-up is restricted to selected hospitals (>1)	5-10%	Any one of the above	Ane one of the above
Low	Methods to specifically control for confounding (eg., instrumental variable approach)	None of the above	None of the above	Follow-up is not limited by specific hospital (eg., Medicare population)	<5%	None of the above	None of the above

© 2025 Balasubramanian I et al. *JAMA Network Open*.

eTable 1: Search strategy
(A) Pubmed

1	"outpatient follow-up"[Title/Abstract] OR "early follow-up"[Title/Abstract] OR "physician follow-up"[Title/Abstract] OR "post-discharge follow-up"[Title/Abstract] OR "discharge follow-up"[Title/Abstract] OR "follow-up post-discharge"[Title/Abstract] OR "ambulatory follow-up"[Title/Abstract] OR "timely follow-up"[Title/Abstract] OR "hospital follow-up"[Title/Abstract] OR "close follow-up"[Title/Abstract] OR "follow-up visit*"[Title/Abstract] OR "physician visit"[Title/Abstract:~2] OR "outpatient visit"[Title/Abstract:~2] OR "physician visit*"[Title/Abstract] OR "outpatient visit*"[Title/Abstract] OR "clinic visit*"[Title/Abstract] OR "office visit*"[Title/Abstract] OR "face-to-face visit*"[Title/Abstract] OR "ambulatory visit*"[Title/Abstract] OR "hospital visit*"[Title/Abstract] OR "ambulatory care"[Title/Abstract] OR "outpatient care"[Title/Abstract] OR "follow-up care"[Title/Abstract] OR "after care"[Title/Abstract] OR "outpatient management"[Title/Abstract] OR "disease management"[Title/Abstract] OR "ambulatory management"[Title/Abstract] OR "follow-up clinic"[Title/Abstract:~7] OR "follow-up within"[Title/Abstract:~2]
2	"readmi*"[Title/Abstract] OR "re-admi*"[Title/Abstract] OR "rehospital*"[Title/Abstract] OR "re-hospital*"[Title/Abstract] OR "subsequent admission"[Title/Abstract:~2] OR "hospitalization within"[Title/Abstract:~2] OR "admission within"[Title/Abstract:~2] OR "hospitalized within"[Title/Abstract:~2] OR "admitted within"[Title/Abstract:~2] OR "patient readmission"[MeSH Terms]
3	#1 AND #2
4	child*[Title/Abstract] OR infant*[Title/Abstract] OR Pediatri*[Title/Abstract] OR paediatr*[Title/Abstract] OR schizophren*[Title/Abstract] OR mental*[Title/Abstract] OR psychiatr*[Title/Abstract] OR postpartum[Title/Abstract] OR obstetr*[Title/Abstract]
5	#3 NOT #4
6	("case reports"[Publication Type] OR "Comment"[Publication Type] OR "Editorial"[Publication Type] OR "Interview"[Publication Type] OR "Letter"[Publication Type] OR "personal narrative"[Publication Type])
7	#5 NOT #6
8	#5 NOT #6: Filters: from 2000 - 3000/12/12

(B) Embase

1	((outpatient near/2 follow-up):ti,ab,kw OR (early near/2 follow-up):ti,ab,kw OR (physician near/2 follow-up):ti,ab,kw OR (post-discharge near/2 follow-up):ti,ab,kw OR (discharge near/2 follow-up):ti,ab,kw OR (follow-up near/2 post-discharge):ti,ab,kw OR (ambulatory near/2 follow-up):ti,ab,kw OR (timely near/2 follow-up):ti,ab,kw OR (hospital near/2 follow-up):ti,ab,kw OR (close near/2 follow-up):ti,ab,kw OR (clinic near/2 follow-up):ti,ab,kw OR 'follow-up visit*':ti,ab,kw OR (physician near/2 visit):ti,ab,kw OR (outpatient near/2 visit):ti,ab,kw OR 'physician visit*':ti,ab,kw OR 'outpatient visit*':ti,ab,kw OR 'clinic visit*':ti,ab,kw OR 'office visit*':ti,ab,kw OR 'face-to-face visit*':ti,ab,kw OR 'ambulatory visit*':ti,ab,kw OR 'hospital visit*':ti,ab,kw OR 'ambulatory care':ti,ab,kw OR 'outpatient care':ti,ab,kw OR 'follow-up care':ti,ab,kw OR 'after care':ti,ab,kw OR 'outpatient management':ti,ab,kw OR 'disease management':ti,ab,kw OR 'ambulatory management':ti,ab,kw OR (follow-up near/7 clinic):ti,ab,kw OR (follow-up near/2 within):ti,ab,kw) AND [english]/lim AND [embase]/lim AND [2000-2025]/py

© 2025 Balasubramanian I et al. *JAMA Network Open*.

2	('readmi*':ti,ab,kw OR 're-admi*':ti,ab,kw OR 'rehospital*':ti,ab,kw OR 're-hospital*':ti,ab,kw OR (subsequent near/2 admission):ti,ab,kw OR (hospitalization near/2 within):ti,ab,kw OR (admission near/2 within):ti,ab,kw OR (hospitalized near/2 within):ti,ab,kw OR (admitted near/2 within):ti,ab,kw OR 'hospital readmission'/exp OR 'hospital readmission') AND [english]/lim AND [embase]/lim AND [2000-2025]/py
3	#1 AND #2
4	#3 AND ('Article'/it OR 'Article in Press'/it OR 'Review'/it)
5	#4 AND ([adult]/lim OR [aged]/lim OR [middle aged]/lim OR [very elderly]/lim OR [young adult]/lim)

(C) CINAHL

S1	XB ((outpatient n3follow-up) OR "early n3 follow-up" OR "physician n3 follow-up" OR "post-discharge n3 follow-up" OR "discharge n3 follow-up" OR "follow-up n3 post-discharge" OR "ambulatory n3 follow-up" OR "timely n3 follow-up" OR "hospital n3 follow-up" OR "close n3 follow-up" OR "follow-up n3 visit*" OR "physician n3 visit*" OR "outpatient n3 visit*" OR "clinic n3 visit*" OR " office n3 visit*" OR "face-to-face n3 visit*" OR "ambulatory n3 visit*" OR "hospital n3 visit*" OR "ambulatory care" OR "outpatient care" OR "follow-up care" OR "after care" OR " outpatient management" OR "disease management" OR "ambulatory management" OR "follow-up n8 clinic" OR "follow-up n3 within")Limiters - Publication Date: 20000101-; English Language; Peer Reviewed; Age Groups: All Adult
S2	XB ("readmi*" OR "re-admi*" OR "rehospital*" OR "re-hospital*" OR "subsequent n3 admission" OR "hospitalization n3 within" OR "admission n3 within" OR "hospitalized n3 within" OR "admitted n3 within") Limiters - Publication Date: 20000101-; English Language; Peer Reviewed; Age Groups: All Adult
S3	MH readmission Limiters - Publication Date: 20000101-; English Language; Peer Reviewed; Age Groups: All Adult
S4	S2 OR S3
S5	S4 AND S1

© 2025 Balasubramanian I et al. *JAMA Network Open*.

eAppendix 2: Formulas used for effect sizes

To calculate crude risk ratio (CRR)

CRR = $\frac{P_1}{P_0}$

where P_1- proportion of people who got readmitted in the ‘follow-up’ group

P_0- proportion of people who got readmitted in the ‘no follow-up’ group

Confidence interval (CI) for CRR

Lower 95% CI =

Upper 95% CI $= e^{\ln(CRR)+1.96\sqrt{\frac{1}{a}+\frac{1}{c}-\frac{1}{a+b}-\frac{1}{c+d}}}$

Where a – number who got redmitted in the ‘follow-up’ group

c – number who got readmitted in the ‘no follow-up’ group

a+b – number who received follow-up

c+d – number who did not receive follow-up

To convert odds ratio (OR) to risk ratio (RRR)

RRR $= \frac{OR}{(1-P_0)+(P_0*OR)}$

The same formula is applied to the 95% confidence interval of odds ratio to obtain 95% confidence interval for relative risk ratio.

© 2025 Balasubramanian I et al. *JAMA Network Open*.

eAppendix 3: Assessing readmission risk in good-quality studies

We assessed the readmission risk of the study samples for all good-quality studies. When available, we used composite scores such as LACE score (incorporating Length of stay, Acuity of admission, Co-morbidities, no of Emergency department visits within the last 6 months) or other scores. Based on standard LACE risk stratification cutoffs (0–4 = low; 5–9 = medium; ≥10 = high), we classified each study sample's average risk level as low, medium or high according to its reported mean LACE score. One study used a raw risk score based on a prediction model4 which indicates the predicted readmission probability (<0.068 – low risk, 0.068-0.10 -medium risk; >0.10- high risk)

Where the LACE score was not reported, we estimated the risk profile using a combination of length of stay (LOS) and comorbidity indices. For LOS, we classified the samples as high risk if the mean or median LOS was at least 5 days; otherwise, they were classified as low risk.

For comorbidity measures, we applied the following criteria:

- Charlson Comorbidity Index (CCI), Charlson/Deyo Comorbidity Index (DCI), or Elixhauser Comorbidity Index (ECI): The samples were classified as high risk if more than 50% of participants had a score greater than 1[1].
- Elixhauser Comorbidity Score (ECS): A mean score of ≥6 was considered high risk[1].
- Hierarchical Condition Category (HCC) score[2]: A mean score of >2 was considered high risk.

Using the above risk classification based on LOS and co-morbidity score, the sample was then stratified into three categories:

- Low risk: low risk on both LOS and comorbidity index
- Medium risk: low risk on one measure and high risk on the other
- High risk: high risk on both measures

© 2025 Balasubramanian I et al. *JAMA Network Open*.

Author, year	Length of stay[a]	Comorbidities[b]	Composite score	Readmission Risk rating
Tung et al,[5] 2017	≥5 days:<br>AMI: 59.3%<br>HF: 58.2%	Charlson Comorbidity Index >1:<br>AMI: 56.4%<br>HF: 71.7%		AMI: High<br>HF: High
Tak et al,[6] 2019	≥5 days:<br>HF: 41.3%<br>AMI:36.4%<br>Pneumonia:42.9%	Charlson Comorbidity Index: Mean (SD)<br>HF: 3.3 (1.8)<br>AMI: 2.9(1.8)<br>Pneumonia: 2.2(1.9)		HF: Medium<br>AMI: Medium<br>Pneumonia: Medium
Anderson et al,[7] 2022	≥5 days:<br>HF: 33.3%<br>AMI: 24%<br>COPD: 27.9%<br>Pneumonia: 31.7%	Hierarchical Condition Category risk score (median, IQR):<br>HF: 2.3 (1.3, 3.8)<br>AMI: 1.0 (0.6,1.9)<br>COPD: 1.9 (1.0, 3.15)<br>Pneumonia: 1.7 (1.0,3.2)		HF: Medium<br>AMI: Low<br>COPD: Low<br>Pneumonia: Low
Saxena et al,[8] 2022	Mean (SD):<br>6.64 (4.82)	Not available		Not available
Lee et al,[12] 2016	Median: 3	Laboratory-based acute physiology(LAP) score[c]:<br>Median -19		Low
Bricard et al,[16] 2019	Mean (SD):<br>9.4(7.1)	Charlson Comorbidity Index:<br>Mean (SD): 1.6(1.7)		High
Baecker et al,[17] 2020			LACE: Mean (SD) –<br>10.8(2.7)	High
Sharma et al,[21] 2010	≥5 days: 42.5%	Elixhauser Comorbidity Index >1:<br>63%		Medium
Terman et al,[33] 2018	Mean (SD):<br>5.2(5.6)	Not available		Not available
Lumpkin et al,[45] 2020	Median (IQR):<br>5(3-7)	Charlson Comorbidity Index:<br>Median (IQR): 1(0-1)		Medium
Moneme et al,[48] 2023	Mean (SD):<br>5.7 (5.6)	Elixhauser Comorbidity Index >1:<br>79.8%		High
Kanwal et al,[49] 2016	≥5 days:<br>41.6%	Charlson/Deyo Comorbidity Index >1:<br>45%		Low
Schletzbaum et al,[50] 2023	Mean (SD):<br>4.6 (4.3)	Hierarchical Condition Category risk score:<br>Mean (SD): 3.5(2.4)		Medium

© 2025 Balasubramanian I et al. *JAMA Network Open*.

Field et al,[63] 2014	≥5 days: 24.3%	Charlson Comorbidity Index>1: 76.3%		Medium
Tong et al,[74] 2018	Not available		Raw risk score[d]: Mean (SD)- 0.15(0.11)	High
Nguyen et al,[78] 2021			LACE: Mean (SD) – 10.4 (2.8)	High
Tak et al,[80] 2021	≥5 days: 34.6%	Charlson Comorbidity Index: Mean (SD) – 1.9(1.9)		Medium
Boggs et al,[84] 2024	≥5 days: 37.7%	Elixhauser Comorbidity Score: Mean (SD)-10.2(8.0)		Medium

Abbreviations: HF – Heart Failure; COPD – Chronic Obstructive Pulmonary Disease; AMI – Acute Myocardial Infraction; LACE – Length of stay, Acuity of admission, Comorbidities, Emergency department visits in last 6 months; SD-standard deviation; IQR – Inter Quartile Range

[a] Numbers in the column indicate the percent of sample who had a length of stay of ≥5 days during their index admission, unless Mean (SD) or Median[IQR] is indicated

[b] Numbers in the column indicate percent of sample whose co-morbidity index is above the indicated value, unless otherwise indicated.

[c] LAP (Laboratory-based acute physiology) score[3] is a severity-of-illness scoring system derived from laboratory data available at hospital admission. The authors note that the LAP score for the sample is low.

[d] The authors use a raw risk score based on a prediction model[4] which indicates the predicted readmission probability (<0.068 – low risk, 0.068-0.10 -medium risk; >0.10- high risk)

© 2025 Balasubramanian I et al. *JAMA Network Open*.

eTable 2: Summary of included studies

Author, year	Sample description				Intervention: Outpatient Follow-up			Outcome/rate[g]	
	Country	Diseases	Sample size	Proportion with age≥65[a]	Time/rate[d]	Provider[f]	Additional components	Readmission	Other outcomes
Tung et al,[5] 2017	Taiwan	Acute Myocardial Infraction (AMI), Heart Failure (HF)	Total: 18585<br>AMI: 5008<br>HF: 13577	AMI: 60.5<br>HF: 75.2	7d: HF-74.9<br>AMI-76.7<br>14d: HF-89.8<br>AMI-93.1	Any physician*, cardiologist		All-cause:<br>HF-23.3<br>AMI-19.9<br>Cardiac related:<br>HF-20.9<br>AMI -18.7	
Tak et al,[6] 2019	US	Heart Failure, Acute Myocardial Infraction, Pneumonia	HF: 30473<br>AMI: 16418<br>Pneumonia : 23046	100	30d[†]: HF-64.0<br>AMI-59.0<br>Pneumonia-60.0	Any physician (institutional), Primary care physician (office), specialist (office), non-physician		All-cause:<br>HF- 17.6<br>AMI-13<br>Pneumonia - 12.4	
Anderson et al,[7] 2022	US	Heart Failure, Acute Myocardial Infraction, Pneumonia, Chronic Obstructive Pulmonary Disease (COPD)	Total: 749402<br>HF: 263746<br>AMI: 115302<br>COPD: 172013<br>Pneumonia : 198341	HF:86.7<br>AMI: 87<br>COPD: 80<br>Pneumonia : 83.5	7d: HF-45.5<br>COPD-40.1<br>AMI-43.7<br>Pneumonia -44.1		Home visits, telehealth	All-cause:<br>HF -19.6<br>COPD-15.6<br>AMI-13<br>Pneumonia - 14.1	
Saxena et al,[8] 2022	Canada	Acute Myocardial Infraction, Heart Failure, Chronic Obstructive Pulmonary Disease	Total: 450746<br>AMI: 198854<br>HF: 133058<br>COPD: 118834	AMI: 52.6<br>HF: 82.2<br>COPD: 74.3	7d: HF-42.5<br>COPD-33.8<br>AMI-45.9	Any physician	Telephone call	All-cause:<br>HF-13.6<br>COPD-10.4<br>AMI-6.9<br>Specific cause:<br>HF-6.0<br>COPD-4.5<br>AMI-2.5	Mortality:<br>HF-2.3<br>COPD-1.7<br>AMI-0.7
Hernandez et al,[9] 2010[b]	US	Heart Failure	30136	100	7d: Not applicable[b]	Any physician (hospital level), specialist		Not applicable[b]	

© 2025 Balasubramanian I et al. *JAMA Network Open*.

Muus et al,[10] 2010	US	Heart Failure	32,998	64.3	30d[†]: 48.0	Physician/physician extender Primary care clinic Cardiology clinic		17.3	
Tuso et al,[11] 2014	US	Heart Failure	2076	Not available	7d: 47.9		Home health visit, telephone call	18.6	
Lee et al,[12] 2016	US	Heart Failure	9522	M (SD): 76.4 (12.0)	30d: Not applicable[e]	Internal medicine, family medicine or cardiology providers	Telephone calls	Not applicable[e]	
McAlister et al,[13] 2016	Canada	Heart Failure	39249	M (SD): 76.1 (12.8)	14d: 65.7	Any physician		All-cause: 17.2 Cardiac-related: 7.5	Mortality: 3.1 ED visits: 30.9 Readmission (all-cause) and mortality: 19.0
Murtaugh et al,[14] 2017	US	Heart Failure	98,730	M (SD) 79.9 (10.3)	7d: 36.9		Home health nursing	20.8	
Huynh et al,[15] 2018	Australia	Heart Failure	906	M (SD): 72.5 (13.9)	7d: 27.6	Cardiologist, GP or HF nurse		17-33	Mortality: 0-13
Bricard et al,[16] 2019	France	Heart Failure	28,848	100	7d: 47.7	Any physician, Primary care physician*		23.9	
Baecker et al,[17] 2020	US	Heart Failure	26128	M(SD): 72.9 (13.5)	7d: 72.5	Any physician/nurse practitioner	Home Health visit, telephone call	18.1	
Distelhorst et al,[18] 2022	US	Heart Failure	1280	Median (IQR): 79 (73,94)	14d: 60.1	Primary care physician		13	
Xu et al,[19] 2022	US	Heart Failure	6918	Median (IQR): 69 (19)	7d: Not reported 14d: 41.9	Any physician from family medicine, internal	Telemedicine visit	19.3	Readmission and Mortality: 20.4

© 2025 Balasubramanian I et al. *JAMA Network Open*.

						medicine, geriatrics or cardiology			
Balasubramanian et al,[20] 2024	Singapore	Heart Failure	1595	52.7	7d: 8.5<br>14d: 17.6<br>21d: 23.7<br>30d: 29.2	Any physician or rehabilitative services		All-cause: 31.9<br>Cardiac cause: 28.7	Readmission (all-cause) and ED visits and mortality: 35.1<br>Readmission (cardiac-cause) and ED visits and mortality: 32.7
Sharma et al,[21] 2010	US	Chronic Obstructive Pulmonary Disease	62,746	100	30d: 66.9	Primary care physician/pulmonologist		18.4	
Fidahussein et al,[22] 2014	US	Chronic Obstructive Pulmonary Disease	1422	M (SD): 66 (17)	30d: 68.4	Primary care physician/pulmonologist		18.9	ED visits: 7.4<br>Mortality: 3.0<br>Readmissions and ED visits: 26.3<br>Readmissions and ED visits and mortality: 29.3
Sharif et al,[23] 2014	US	Chronic Obstructive Pulmonary Disease	8263	M (SD): 56.55 (5.73)	30d: 57.3			9	
Hijjawi et al,[24] 2015	US	Chronic Obstructive Pulmonary Disease	192	M (SD): 65.8 (12.8)	30d: 62	Primary care physician		19.4	
Russo et al,[25] 2017	US	Chronic Obstructive Pulmonary Disease	160	M (SD): 65.9 (10.0)	7d: 73.8	COPD exacerbation clinic (mid-level provider, physician, educator)	Care co-ordination	18.1	
Budde et al,[26] 2019	US	Chronic Obstructive Pulmonary Disease	2563	Not available	10d: 24.3	Primary care physician or any medical subspecialist		17.6	

© 2025 Balasubramanian I et al. *JAMA Network Open*.

Jiang et al,[27] 2024	Canada	Chronic Obstructive Pulmonary Disease	94,034	Median (IQR): 74.0 (64.0– 82.0)	30d: 73.5	Any physician Pulmonologist		Not reported	
Hess et al,[28] 2013[b]	US	Acute Myocardial Infraction	25872	100	7d: Not applicable[b]	Any physician (hospital level)		Not applicable[b]	
Brown et al,[29] 2014[b]	US	Acute Myocardial Infraction	188611	100	14d: Not applicable[b]	Any physician (hospital level)		Not applicable[b]	
Zabawa et al,[30] 2018	France	Acute Myocardial Infraction	624	100	7d: 53.0	GPs, cardiologists, endocrinologists		23	
Condon et al,[31] 2016	US	Stroke	510	M (SD): 65.1(13.2)	7-14d: 74.9	Nurse practitioner-led transitional stroke clinic		9	
Allen et al,[32] 2017	US	Stroke	416	M: 70.8	21d: 30.5	Neurologist		13.7	
Terman et al,[33] 2018	US	Stroke	78,345	M (SD): 77.8 (8.3)	30d: 77.1	Any physician*, Primary care physician, neurologist		9.4	
Leppert et al,[34] 2020	US	Stroke	14630	M(SD): 62.9 (12.0)	30d: 83.7	Any physician*, Primary care physician neurologist		7.3	
Hussein et al,[35] 2022	US	Stroke	872	M(SD): 71.2 (14)	30d: 51.8	Primary care physician		10.1	ED visits: 8.9 Readmissions and ED visits: 19.0
Bennett et al,[36] 2012	US	Diabetes	21275	100	30d: 84.6	Any physician		14.4	
Karunakaran et al,[37] 2018	US	Diabetes	44203	44.75	30d: 45.3			20.4	
Rubin et al,[38] 2023[c]	US	Diabetes	17284	37.4	30d: Not reported			Not reported	
Johnson et al,[39] 2013	US	Atrial fibrillation	4228	M (SD): 73.4 (11.7)	7d: 39.6	Cardiologist, Primary care physician, clinician, other		13.6	

© 2025 Balasubramanian I et al. *JAMA Network Open*.

						specialists, INR testing, prescription for anticoagulant			
Hubbard et al,[40] 2014	US	Atrial fibrillation, Chronic diseases	AF:148020 Chronic:182683	AF: 95.1 chronic: 86.7	14d: AF-50.9 chronic-47.4	Any physician		AF-10.9 chronic-9.4	
Brooke et al,[41] 2014	US	Aneurysm surgery, Hernia surgery	Aneurysm: 2437 Hernia: 52807	100	30d: Aneurysm-75.8 Hernia-11.1	Primary care physician	Home visit	Aneurysm-22.1 Hernia-9.3	
Saunders et al,[42] 2014	US	General surgery, Vascular surgery	General: 7752 Vascular: 2362	General: 53.5 (24) Vascular: 65(19)	30d: General-84.1 Vascular-75.3	Primary care physician/specialist/surgeon		General-10.3 Vascular-9.8	
Manji et al,[43] 2017	Canada	Cardiac surgery	9210	Median (IQR): 72 (62–77)	30d: 87.4	Any physician		18.3	
Poulose et al,[44] 2018	US	Hernia surgery	3007	M(SD): 55.65 (13.67)	30d: 29.9		Questionnaire	4.7	
Lumpkin et al,[45] 2020	US	Colorectal surgery	3442	M (SD): 59 (7)	10d: 37.8			28.3	ED visits: 11.4
Ghiam et al,[46] 2022	US	Endoscopic Transphenoidal Pituitary surgery	409	M (SD): 53.9 (16.5)	30d: 68.5	Endocrinologist		13.9	
Fair et al,[47] 2023	US	Laproscopic cholecystectomy	661	Median (IQR): 44.0 (32.0-55.0)	30d: 67.9			6.4	ED visits: 18.4
Moneme et al,[48] 2023	US	General surgery	345360	74.4 (12.0)	30d: 45.4	Primary care physician		16.9	
Kanwal et al,[49] 2016	US	Cirrhosis	25217	M (SD): 62.0 (8.9)	7d: 32.2	Any clinician		13.8	Mortality: 4.7
Schletzbaum et al,[50] 2023	US	Lupus	8606	38.6	14d: Not reported 30d: 52.1	Primary care physician/rheumatologist		22.4	ED visits: 34.2 Mortality: 1.3 Readmission and ED visits: 61.5
Leschke et al,[51] 2012	US	Sickle cell disease	408	0.25	30d: 37.3			17.2	

© 2025 Balasubramanian I et al. *JAMA Network Open*.

Hazratjee et al,[52] 2013	US	Irritable Bowel Disease	539	M (SD): 42.7 (16.2)	30d: 45.5	Gastroenterologist		18	
Sbeit et al,[53] 2021	Israel	Irritable Bowel Disease	176	M(SD): 37.6 (17.7)	30d: 72.2	Gastroenterologist		9.7	
Berry et al,[54] 2013	US	Human Immunodeficiency Virus (HIV)	11651	M (SD): 44 (38 - 51)	30d: 66.3			19.3	
Hill et al,[55] 2024	US	Human Immunodeficiency Virus (HIV)	114	Median [IQR]: 51 [40,61] 48 [41,56]	30d: 78.9	HIV discharge clinic: HIV specialist physician, social workers, behavioural health counsellors, HIV pharmacists		13.2	
Saini et al,[56] 2019	US	Outpatient Parenteral Antimicrobial Therapy (OPAT) patients	388	M: 59.6	14d: Not applicable[e]	OPAT clinic		Not applicable[e]	
Palms et al,[57] 2020	US	Outpatient Parenteral Antimicrobial Therapy (OPAT) patients	755	Median [IQR]: 58 [45,67]	30d: 72.6	OPAT clinic (physicians, advanced practice provider, registered nurse and a medical assistant)		18.1	
Dalton et al,[58] 2017	US	Trauma patients	2134	M (SD): 41.9 (17.1) 53.7 (21.6)	30d: 10.3	Trauma clinic		4.9	ED visits: 13.1
Smith et al,[59] 2017	US	Trauma patients	2266	Median [IQR]: 48 [31-65]	30d: 75.8	Any health care professional		4.1	
Deb et al,[60] 2019	US	Sepsis	170571	M (SD): 76 (12.6)	7d: 39.1		Home health nursing	16.1	
Blank et al,[61] 2025	US	Epilepsy/seizure	80620	100	30d: 29.3	Primary care physician/specialist (neurology,		17.7	

© 2025 Balasubramanian I et al. *JAMA Network Open*.

						neurosurgery /epilepsy)			
Misky et al,[62] 2010	US	General inpatients	65	62.4	30d: 49.2	Primary care physician		Same cause: 12.3 Any cause: 12.5	Readmission and ED visits (same cause): 16.9
Field et al,[63] 2014	US	General inpatients	3661	100	7d: 49.4	Primary care physician, any physician*		19.3	
Jackson et al,[64] 2015[c]	US	General inpatients	44473	M (SD): 26.5 (21.1)	7d/14d/21d/30d: Not reported	Primary care physician or specialist		Not reported	
Bennett et al,[65] 2016	US	General inpatients	297084	84.1	30d: 66.3	Any physician		16.9	
Wang et al,[66] 2016	US	General inpatients	55532	M(SD): 49.3 (15.1)	30d: 40.0			10.8	
Chakravarthy et al,[67] 2018	US	General inpatients	280	M(SD): 49.4(14.6) 49.1(16.2)	7d: 45.4	Transition clinic (Primary care physician, registered nurse, social worker and a patient navigator)		15.0	
Sinha et al,[68] 2017	US	General inpatients	3613	M(SD): 55.31 (17.91)	14d: 24.1	Primary care physician		11.1	
Shen et al,[69] 2017	US	General inpatients	71231	M(SD): 75(9)	7d: 25.6	Dedicated post-hospital follow-up provider clinic visit*/Any visit		10.2	Mortality: 0.9
Toth et al,[70] 2017[c]	US	General inpatients	11492	100	14d: Not reported			11.3	ED visits: 6.7 Mortality: 1.4
Ballard et al,[71] 2018	US	General inpatients	1884	52.9 (16)	14d: 51.2		Telephone call	3.7	

© 2025 Balasubramanian I et al. *JAMA Network Open*.

Hawes et al,[72] 2018	US	General inpatients	268	57.2 (15.7)	7d: 43.3<br>14d: 67.2<br>30d: 84	Primary care physician		17.9	
Lam et al,[73] 2018	Canada	General inpatients	214	M(SD): 70 (16)	7d: 57.9	Primary care physician			Readmissions and ED visits: 30.8
Tong et al,[74] 2018	US	General inpatients	38068	Not available	30d: 47.7	Any physician		13	
Rayan-Gharra et al,[75] 2019	Israel	General inpatients	594	M(SD): 60.90 (17.74)	14d: 93.6	Primary care physician		17	
Wiest et al,[76] 2019	US	General inpatients	1531	Not available	7d: 29.4	Primary care physician		15.3	
Baldino et al,[77] 2021	US	General inpatients	1373	M: 57.1	14d: 27.2	Transition clinic (hospitalist, registered nurse, a social worker and a patient navigator)		13.5	
Nguyen et al,[78] 2021	US	General inpatients	134507	M (SD): 60.9 (18.1)	7d: 26.4	Dedicated post-hospital follow-up provider clinic visit*/Any clinic visit		13.5	
Patel et al,[79] 2021	US	General inpatients	3296	Not available	14d: Not applicable[e]		Telephone call and non face-to-face services	Not applicable[e]	
Tak et al,[80] 2021	US	General inpatients	583199	100	7d[†]: 36.0<br>14d[†]: 45.0<br>30d[†]: 54.0	Any physician(institu tional),<br>Primary care physician (office),<br>specialist (office),<br>non-physician		11.8	

Van de Graff et al,[81] 2021[c]	US	General inpatients	574	M(SD): 64.1(14.4)	7d: 61.5	Transition care clinic		Not reported	
Kojima et al,[82] 2022	US	General inpatients	9236	57.9 (20.1)	7d: 24.1 14: 35.6	Primary care physician		3.6	
Schaub et al,[83] 2022	Canada	High-risk patients	2119	71.1 (15.6)	14d: 83.8	Primary care physician		17.2	ED visits: 28.0
Boggs et al,[84] 2024	US	Sepsis, diabetes, Heart failure, kidney and liver disease	4281	0	30d: 71.4			Not reported	
Brady et al,[85] 2024	US	Chronic diseases	1118	62.3 (15.5)	30d: 63.2	Primary care physician/specia list	Telehealth visits	18.2	
Naseer et al,[86] 2024	Sweden	General inpatients	6135	82.9 (8.1)	30d: Not reported			12.8	
Sass et al,[87] 2024	US	General inpatients	727	M(SD): 53.5 (15.8)	7-14d: 62.2	Discharge clinic (1 physician, 1 nurse practitioner, 2 registered nurse, 2 medical assistants and 1 clinical manager)		20.4	

Abbreviations: ED- Emergency Department; M(SD) – Mean (Standard Deviation); IQR – Inter Quartile Range

a- Numbers in the column indicate the percent of sample aged 65 years and above, unless mean (standard deviation) or Median [Inter quartile range) is indicated as M [SD] or Median [IQR]; some papers stratify the 2-demographics by readmission status or follow-up status in which case we present both the values of M(SD) or Median(IQR)

b- Studies excluded from meta-analysis (hospital level studies); Readmission rates and follow-up rates are not applicable

c – Studies excluded from meta-analysis as relevant effect sizes are not available.

d – Time to outpatient follow-up indicates that outpatient follow-up happened within 7,10, 14,21 or 30 days within discharge; The numbers in the column indicate the percentage of sample who received outpatient follow-up in the specified time.

* In case of multiple providers, the numbers indicate the rate of follow-up with the provider marked with * in the 'provider' column (for which the effect size is available/used).

† In some studies, the effect sizes are given for multiple providers. In such cases, we used a pooled fixed-effect estimate in the meta-analysis. For these studies, we report the pooled prevalence value.

e- Studies using case-control design; Readmission rates and follow-up rates are not applicable.

f- Any physician includes both Primary care physician and specialist; Wherever blank, the provider is not mentioned in the study

g- Outcomes list all the outcomes within **30 days** for which either the prevalence or effect sizes are given in the paper.

© 2025 Balasubramanian I et al. *JAMA Network Open*.

eTable 3: Quality Assessment of studies: ROBINS – I

Author, year	Bias due to confounding	Bias in selection of participants	Bias in classification of interventions	Bias due to deviations from intended interventions	Bias due to missing data	Bias in measurement of outcomes	Bias in selection of the reported result	Overall Bias
Tung et al,[5] 2017	Low	Moderate	Low	Low	Low	Moderate	Low	Moderate
Tak et al,[6] 2019	Moderate	Moderate	Low	Low	No Information	Moderate	Low	Moderate
Anderson et al,[7] 2022	Moderate	Low	Moderate	Low	No Information	Moderate	Low	Moderate
Saxena et al,[8] 2022	Low	Moderate	Low	Low	No Information	Low	Low	Moderate
Hernandez et al,[9] 2010[b]	Low	Low	Low	Low	Low	Low	Low	Low
Muus et al,[10] 2010	Serious	Serious	Low	Moderate	Moderate	Serious	Serious	Serious
Tuso et al,[11] 2014	Critical	Moderate	Moderate	Low	No Information	Serious	Low	Critical
Lee et al,[12] 2016	Low	Moderate	Low	Low	Low	Low	Low	Moderate
McAlister et al,[13] 2016	Moderate	Low	Low	Low	No Information	Low	Low	Moderate
Murtaugh et al,[14] 2017	Low	Serious	Low	Low	Low	Moderate	Low	Serious
Huynh et al,[15] 2018	Critical	Moderate	Moderate	Moderate	Serious	Serious	Serious	Critical
Bricard et al,[16] 2019	Low	Moderate	Low	Low	Low	Moderate	Low	Moderate
Baecker et al,[17] 2020	Moderate	Low	Low	Low	Low	Low	Low	Moderate
Distelhorst et al,[18] 2022	Moderate	Low	Moderate	Low	Moderate	Serious	Low	Serious
Xu et al,[19] 2022	Low	Serious	Low	Moderate	Moderate	Serious	Low	Serious
Balasubramanian et al,[20] 2024	Moderate	Moderate	Low	Moderate	No Information	Moderate	Low	Moderate
Sharma et al,[21] 2010	Moderate	Moderate	Moderate	Low	Low	Low	Low	Moderate
Fidahussein et al,[22] 2014	Moderate	Moderate	Low	Serious	No Information	Serious	Low	Serious
Sharif et al,[23] 2014	Moderate	Moderate	Moderate	Low	No Information	Serious	Low	Serious
Hijjawi et al,[24] 2015	Critical	Moderate	Low	Serious	Low	Critical	Moderate	Critical
Russo et al,[25] 2017	Critical	Moderate	Low	Serious	Low	Critical	Moderate	Critical
Budde et al,[26] 2019	Moderate	Moderate	Moderate	Serious	No Information	Critical	Low	Critical

© 2025 Balasubramanian I et al. *JAMA Network Open*.

Jiang et al,[27] 2024	Low	Moderate	Low	Low	Low	Low	Low	Moderate
Hess et al,[28] 2013[b]	Low	Moderate	Low	Low	Low	Low	Low	Moderate
Brown et al,[29] 2014[b]	Low	Moderate	Low	Low	Moderate	Moderate	Low	Moderate
Zabawa et al,[30] 2018	Critical	Serious	Low	Low	Low	Moderate	Low	Critical
Condon et al,[31] 2016	Moderate	Moderate	Low	Serious	Moderate	Critical	Low	Critical
Allen et al,[32] 2017	Critical	Moderate	Low	Serious	No Information	Critical	Low	Critical
Terman et al,[33] 2018	Moderate	Low	Low	Low	Moderate	Low	Low	Moderate
Leppert et al,[34] 2020	Moderate	Serious	Low	Low	Serious	Moderate	Low	Serious
Hussein et al,[35] 2022	Serious	Moderate	Low	Low	No information	Serious	Low	Serious
Bennett et al,[36] 2012	Moderate	Serious	Low	Low	Moderate	Moderate	Low	Serious
Karunakaran et al,[37] 2018	Moderate	Moderate	Low	Serious	Low	Serious	Moderate	Serious
Rubin et al,[38] 2023[c]	Moderate	Moderate	Low	Serious	Low	Serious	Moderate	Serious
Johnson et al,[39] 2013	Moderate	Serious	Low	Low	No Information	Low	Low	Serious
Hubbard et al,[40] 2014	Moderate	Low	Low	Low	No Information	Serious	Moderate	Serious
Brooke et al,[41] 2014	Moderate	Low	Low	Low	No Information	Serious	Moderate	Serious
Saunders et al,[42] 2014	Critical	Moderate	Low	Serious	No Information	Critical	Low	Critical
Manji et al,[43] 2017	Moderate	Low	Low	Low	No information	Serious	Low	Serious
Poulose et al,[44] 2018	Serious	Moderate	Low	Low	Low	Serious	Low	Serious
Lumpkin et al,[45] 2020	Low	Moderate	Low	Low	Low	Moderate	Low	Moderate
Ghiam et al,[46] 2022	Moderate	Low	Low	Serious	No Information	Critical	Low	Critical
Fair et al,[47] 2023	Moderate	Low	Low	Serious	No information	Critical	Low	Critical
Moneme et al,[48] 2023	Low	Low	Low	Low	Low	Moderate	Low	Moderate
Kanwal et al,[49] 2016	Low	Moderate	Low	Low	Low	Low	Low	Moderate
Schletzbaum et al,[50] 2023	Moderate	Low	Low	Low	No Information	Low	Low	Moderate
Leschke et al,[51] 2012	Moderate	Low	Low	Serious	No information	Critical	Low	Critical
Hazratjee et al,[52] 2013	Critical	Low	Low	Serious	Low	Critical	Low	Critical
Sbeit et al,[53] 2021	Critical	Moderate	Low	Moderate	No Information	Critical	Moderate	Critical
Berry et al,[54] 2013	Moderate	Moderate	Low	Low	Moderate	Serious	Low	Serious

© 2025 Balasubramanian I et al. *JAMA Network Open*.

Hill et al,[55] 2024	Moderate	Moderate	Moderate	Serious	No information	Critical	Low	Critical
Saini et al,[56] 2019	Low	Moderate	Low	Serious	Low	Critical	Low	Critical
Palms et al,[57] 2020	Moderate	Moderate	Low	Moderate	Moderate	Critical	Low	Critical
Dalton et al,[58] 2017	Moderate	Moderate	Low	Serious	No Information	Critical	Low	Critical
Smith et al,[59] 2017	Moderate	Serious	Low	Serious	Moderate	Serious	Low	Serious
Deb et al,[60] 2019	Low	Serious	Low	Low	Low	Moderate	Low	Serious
Blank et al,[61] 2025	Moderate	Low	Low	Low	Low	Low	Low	Moderate
Misky et al,[62] 2010	Serious	Moderate	Moderate	Serious	No information	Critical	Moderate	Critical
Field et al,[63] 2014	Low	Moderate	Low	Moderate	No Information	Low	Low	Moderate
Jackson et al,[64] 2015[c]	Serious	Moderate	Low	Low	No Information	Moderate	Moderate	Serious
Bennett et al,[65] 2016	Moderate	Moderate	Moderate	Low	No Information	Moderate	Low	Serious
Wang et al,[66] 2016	Critical	Moderate	Moderate	Serious	No Information	Critical	Low	Critical
Chakravarthy et al,[67] 2018	Moderate	Moderate	Low	Serious	No Information	Critical	Moderate	Critical
Sinha et al,[68] 2017	Moderate	Moderate	Low	Serious	Low	Serious	Low	Serious
Shen et al,[69] 2017	Moderate	Low	Moderate	Low	Low	Low	Low	Moderate
Toth et al,[70] 2017[c]	Low	Serious	Low	Low	Low	Moderate	Low	Serious
Ballard et al,[71] 2018	Serious	Moderate	Moderate	Serious	No Information	Critical	Low	Critical
Hawes et al,[72] 2018	Critical	Moderate	Low	Serious	No Information	Critical	Low	Critical
Lam et al,[73] 2018	Moderate	Moderate	Moderate	Moderate	Low	Critical	Low	Critical
Tong et al,[74] 2018	Moderate	Moderate	Low	Low	No Information	Moderate	Low	Moderate
Rayan-Gharra et al,[75] 2019	Moderate	Low	Moderate	Serious	No information	Critical	Low	Critical
Wiest et al,[76] 2019	Low	Serious	Moderate	Low	No Information	Serious	Low	Serious
Baldino et al,[77] 2021	Critical	Moderate	Low	Serious	No Information	Critical	Moderate	Critical
Nguyen et al,[78] 2021	Low	Low	Moderate	Low	Low	Low	Low	Moderate
Patel et al,[79] 2021	Low	Serious	Low	Moderate	Moderate	Serious	Low	Serious
Tak et al,[80] 2021	Moderate	Moderate	Low	Low	Low	Moderate	Low	Moderate
Van de Graff et al,[81] 2021[c]	Critical	Moderate	Low	Serious	No Information	Critical	Moderate	Critical

© 2025 Balasubramanian I et al. *JAMA Network Open*.

Kojima et al,[82] 2022	Moderate	Low	Low	Moderate	Low	Critical	Low	Critical
Schaub et al,[83] 2022	Critical	Serious	Low	Serious	No Information	Critical	Serious	Critical
Boggs et al,[84] 2024	Moderate	Moderate	Low	Low	No Information	Low	Low	Moderate
Brady et al,[85] 2024	Moderate	Serious	Moderate	Serious	No Information	Serious	Moderate	Serious
Naseer et al,[86] 2024	Moderate	Low	Low	Low	Moderate	Low	Low	Moderate
Sass et al,[87] 2024	Serious	Moderate	Low	Serious	No Information	Critical	Moderate	Critical

© 2025 Balasubramanian I et al. *JAMA Network Open*.

eFigure 1: Summary of bias across studies

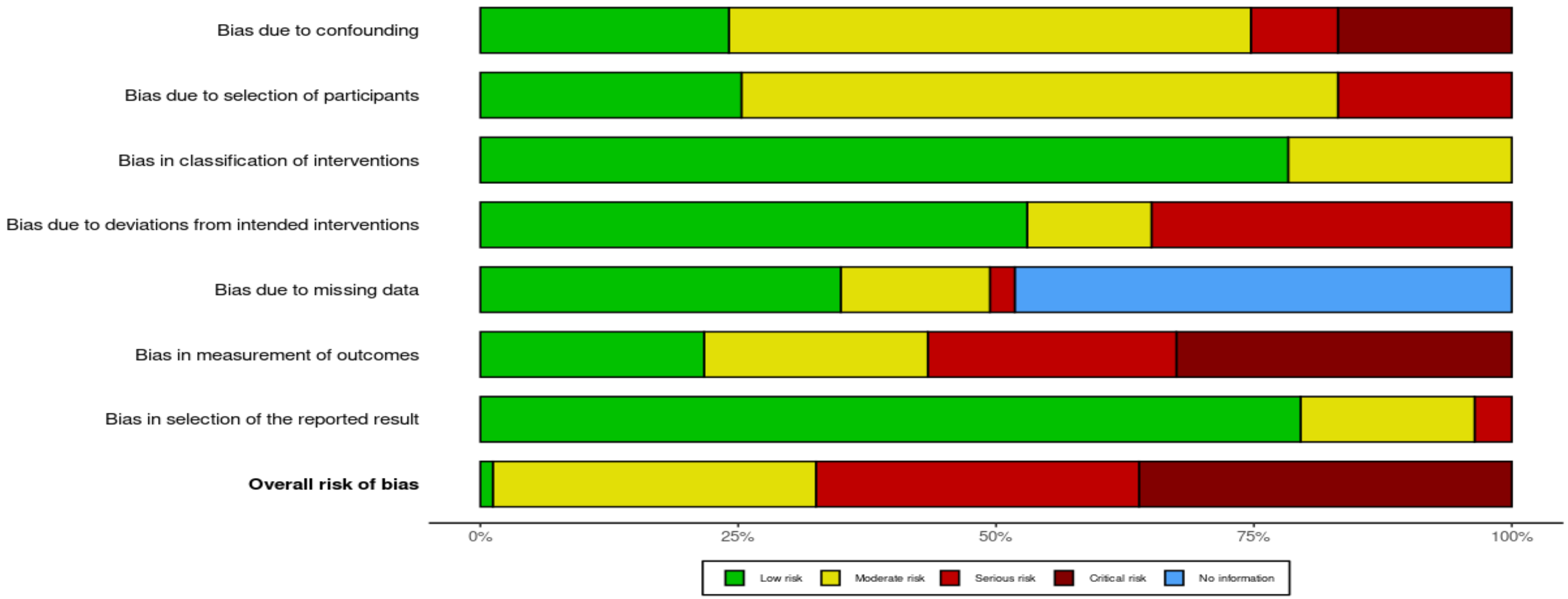

© 2025 Balasubramanian I et al. *JAMA Network Open*.

eTable 4: Meta-analysis results– subgroup analysis by disease, age and time to outpatient follow-up

Time to Outpatient Follow-up	Disease	Sample age group (Mean/median)	No of studies N (%)	No of assessments N (%)	Pooled Relative Risk Ratio (95% CI)	I^2	No of studies N (%)	No of assessments N (%)	Pooled Relative Risk Ratio (95% CI)	I^2
			**ALL STUDIES**				**GOOD QUALITY STUDIES**			
<30 days	All	All	73 (100)	92 (100)	0.68 (0.60,0.75)	99.81	21 (29)	34 (37)	0.78 (0.67,0.89)	99.89
		<65	32 (44)	35 (38)	0.57 (0.46,0.68)	97.85	5 (7)	6 (7)	1.01 (0.89,1.13)	80.49
		>=65	36 (49)	52 (57)	0.76 (0.66,0.85)	99.86	15 (21)	27 (29)	0.71 (0.58,0.83)	99.91
	HF	All (>=65)	14 (19)	16 (17)	0.66 (0.55,0.78)	99.52	7 (10)	8 (9)	0.65 (0.48,0.83)	99.64
	AMI	All (>=65)	5 (7)	6 (7)	0.64 (0.37,0.91)	99.78	4 (5)	5 (5)	0.56 (0.32,0.80)	99.84
	Other diseases	All	61 (84)	70 (76)	0.67 (0.59,0.76)	99.82	17 (23)	21 (23)	0.81 (0.69,0.93)	99.86
		<65	32 (44)	35 (38)	0.57 (0.46,0.68)	97.85	5 (7)	6 (7)	1.01 (0.89,1.13)	80.49
		>=65	25 (34)	31 (34)	0.79 (0.67,0.92)	99.87	11 (15)	14 (15)	0.73 (0.59,0.87)	99.91
<14days	All	All	37 (51)	49 (53)	0.75 (0.67,0.83)	99.72	13 (18)	22 (24)	0.78 (0.63,0.93)	99.89
		<65	12 (16)	13 (14)	0.71 (0.56,0.86)	93.07	4 (5)	4 (4)	0.99 (0.86,1.13)	86.61
		>=65	21 (29)	32 (35)	0.77 (0.66,0.89)	99.85	9 (12)	18 (20)	0.7 (0.53,0.86)	99.91
	HF	All (>=65)	11 (15)	13 (14)	0.68 (0.55,0.82)	99.5	5 (7)	6 (7)	0.63 (0.40,0.87)	99.71
	AMI	All (>=65)	4 (5)	5 (5)	0.67 (0.30,1.03)	99.96	3 (4)	4 (4)	0.57 (0.22,0.91)	99.84
	Other diseases	All	27 (37)	31 (34)	0.77 (0.67,0.87)	99.58	10 (14)	12 (13)	0.84 (0.69,1.06)	99.79
		<65	12 (16)	13 (14)	0.71 (0.56,0.86)	93.07	4 (5)	4 (4)	0.99 (0.86,1.13)	86.61
		>=65	12 (16)	15 (16)	0.83 (0.68,1.04)	99.8	6 (8)	8 (9)	0.76 (0.57,1.05)	99.87
<7days	All	All	21 (29)	27 (29)	0.8 (0.70,0.91)	99.62	10 (14)	16 (17)	0.8 (0.66,0.94)	99.72
		<65	4 (5)	4 (4)	0.82 (0.59,1.04)	94.92	2 (3)	2 (2)	0.96 (0.69,1.22)	96.05
		>=65	15 (21)	21 (23)	0.81 (0.67,0.95)	99.74	8 (11)	14 (15)	0.76 (0.60,0.92)	99.75
	HF	All (>=65)	9 (12)	9 (10)	0.7 (0.55,0.84)	99.38	5 (7)	5 (5)	0.68 (0.47,0.89)	99.51
	AMI	All (>=65)	4 (5)	4 (4)	0.72 (0.41,1.03)	99.59	3 (4)	3 (3)	0.63 (0.34,0.92)	99.68
	Other diseases	All	13 (18)	14 (15)	0.85 (0.72,0.98)	99.56	7 (10)	8 (9)	0.86 (0.69,1.06)	99.66
		<65	4 (5)	4 (4)	0.82 (0.59,1.04)	94.92	2 (3)	2 (2)	0.96 (0.69,1.22)	96.05
		>=65	8 (11)	9 (10)	0.89 (0.70,1.07)	99.82	5 (7)	6 (7)	0.82 (0.62,1.08)	99.77

Abbreviations: HF – Heart Failure; AMI – Acute Myocardial Infraction; CI – Confidence Interval
*Note: 5 studies do not mention the age of the sample (1 HF study and 4 non-HF studies). Hence, the number of assessments for studies with mean/median age >=65 and <65 do not add up to the total number of assessments in the group; There are no HF/AMI studies with mean/median age<65.

© 2025 Balasubramanian I et al. *JAMA Network Open*.

eTable 5: Meta-regression results using Readmission Risk score as a predictor

Subgroup	Readmission Risk categories	Relative Risk ratio (95% Confidence Interval)
Disease: HF/AMI<br>Age: ≥65	Low<br>Medium<br>High	0.64 (0.43, 0.96)<br>0.62 (0.41, 0.92)<br>0.57 (0.37, 1.17)
Disease: Other than HF/AMI<br>Age:≥65	Low<br>Medium<br>High	0.52 (0.33, 0.83)<br>0.82 (0.65, 1.03)<br>0.39 (0.25, 0.63)
Disease: Other than HF/AMI<br>Age:<65	Low<br>Medium<br>High	1.10 (1.02, 1.19)<br>1.08 (1.01, 1.15)<br>0.83 (0.77, 0.90)

Abbreviations: HF – Heart Failure; AMI – Acute Myocardial Infraction;
Notes: See Appendix 3 for details on risk rating. The Hazard ratio and confidence intervals has been calculated by the author using the coefficients of the meta-regression.

© 2025 Balasubramanian I et al. *JAMA Network Open*.

eTable 6: Sensitivity analysis results: Meta-analysis using composite outcome when available

Time to outpatient visit	Disease	Sample age group (Mean/median)	No of studies N (%)	No of assessments N (%)	Pooled Relative Risk Ratio (95% CI)	I^2	No of studies N (%)	No of assessments N (%)	Pooled Relative Risk Ratio (95% CI)	I^2
			ALL STUDIES				GOOD QUALITY STUDIES			
<30 days	All	All	76(100)	98(100)	0.68 (0.61,0.75)	99.79	23(30)	39(40)	0.79 (0.68,0.89)	99.87
		<65	32(42)	35(36)	0.57 (0.46,0.68)	97.99	5(7)	6(6)	1.02 (0.89,1.14)	83.34
		>=65	39(51)	58(59)	0.75 (0.66,0.84)	99.84	17(22)	32(33)	0.72 (0.61,0.84)	99.89
	HF	All (>=65)	15(20)	20(20)	0.66 (0.55,0.77)	99.26	8(11)	12(12)	0.66 (0.51,0.82)	99.11
	AMI	All (>=65)	5(7)	6(6)	0.64 (0.37,0.91)	99.78	4(5)	5(5)	0.56 (0.32,0.80)	99.84
	Other diseases	All	63(83)	72(73)	0.68 (0.59,0.76)	99.83	18(24)	22(22)	0.83 (0.71,0.94)	99.86
		<65	32(42)	35(36)	0.57 (0.46,0.68)	97.99	5(7)	6(6)	1.01 (0.89,1.13)	83.34
		>=65	27(36)	33(34)	0.79 (0.67,0.91)	99.86	12(16)	15(15)	0.75 (0.61,0.89)	99.91
<14days	All	All	40(53)	53(54)	0.76 (0.68,0.83)	99.71	15(20)	25(26)	0.79 (0.66,0.93)	99.88
		<65	12(16)	13(13)	0.71 (0.56,0.86)	93.61	4(5)	4(4)	1 (0.86,1.13)	88.14
		>=65	24(32)	36(37)	0.77 (0.67,0.88)	99.83	11(14)	21(21)	0.73 (0.58,0.88)	99.9
	HF	All (>=65)	12(16)	15(15)	0.68 (0.56,0.81)	99.34	6(8)	8(8)	0.65 (0.45,0.85)	99.5
	AMI	All (>=65)	4(5)	5(5)	0.67 (0.30,1.03)	99.96	3(4)	4(4)	0.57 (0.22,0.91)	99.84
	Other diseases	All	29(38)	33(34)	0.78 (0.68,0.87)	99.6	11(14)	13(13)	0.86 (0.72,1.06)	99.8
		<65	12(16)	13(13)	0.71 (0.56,0.86)	93.61	4(5)	4(4)	1 (0.86,1.13)	88.14
		>=65	14(18)	17(17)	0.83 (0.70,1.03)	99.79	7(9)	9(9)	0.79 (0.61,1.08)	99.88
<7days	All	All	23(30)	29(30)	0.8 (0.70,0.90)	99.57	11(14)	17(17)	0.8 (0.67,0.93)	99.68
		<65	4(5)	4(4)	0.82 (0.59,1.04)	94.92	2(3)	2(2)	0.96 (0.69,1.22)	96.05
		>=65	17(22)	23(23)	0.8 (0.68,0.93)	99.7	9(12)	15(15)	0.76 (0.62,0.91)	99.71
	HF	All (>=65)	10(13)	10(10)	0.7 (0.57,0.84)	99.24	6(8)	6(6)	0.7 (0.51,0.88)	99.27
	AMI	All (>=65)	4(5)	4(4)	0.72 (0.41,1.03)	99.59	3(4)	3(3)	0.63 (0.34,0.92)	99.68
	Other diseases	All	14(18)	15(15)	0.84 (0.72,0.97)	99.51	7(9)	8(8)	0.86 (0.70,1.07)	99.66
		<65	4(5)	4(4)	0.82 (0.59,1.04)	94.92	2(3)	2(2)	0.96 (0.69,1.22)	96.05
		>=65	9(12)	10(10)	0.89 (0.69,1.04)	99.73	5(7)	6(6)	0.82 (0.62,1.11)	99.77

Abbreviations: HF – Heart Failure; AMI – Acute Myocardial Infraction; CI – Confidence Interval

*Note: 5 studies do not mention the age of the sample (1 HF study and 4 non-HF studies). Hence, the number of assessments for studies with mean/median age >=65 and <65 do not add up to the total number of assessments in the group; There are no HF/AMI studies with mean/median age<65.

© 2025 Balasubramanian I et al. *JAMA Network Open*.

eTable 7: Meta-regression results evaluating the effect of statistic used and quality of studies

Variable	Coefficient	Standard error	p-value
Statistic used: Ref: Hazard ratio			
Relative Risk Ratio	-0.14	0.15	0.36
Odds ratio	0.05	0.15	0.73
Crude Risk ratio	-0.03	0.16	0.87
Quality rating: Ref: Moderate			
Serious	0.01	0.15	0.96
Critical	-0.41	0.16	0.01

Notes: The coefficients denote the change in log of effect size (pooled risk ratio (RR)) for the given category compared to the reference category. A negative value indicates that the log RR and RR are lower for studies belonging to the given category compared to the reference category.

© 2025 Balasubramanian I et al. *JAMA Network Open*.

eTable 8: Meta-regression results evaluating the effect of quality domains and specific biases

(A) Bias across domains

Bias domain		Coefficient	Standard error	p-value
Confounding	Ref: Low			
	Moderate	0.18	0.15	0.22
	Serious	-0.23	0.24	0.33
	Critical	-0.10	0.20	0.63
Selection	Ref: Low			
	Moderate	0.18	0.13	0.15
	Serious	0.62	0.18	0.00
Classification of Intervention	Ref: Low			
	Moderate	-0.10	0.12	0.38
Deviation from intended intervention	Ref: Low			
	Moderate	-0.43	0.22	0.05
	Serious	-0.24	0.21	0.27
Missing data	Ref: Low			
	Moderate	0.06	0.18	0.75
	Serious	-0.14	0.34	0.67
	No information	0.06	0.12	0.60
Measurement of outcome	Ref: Low			
	Moderate	-0.35	0.16	0.03
	Serious	-0.37	0.16	0.02
	Critical	-0.40	0.25	0.11
Reported result	Ref: Low			
	Moderate	0.10	0.16	0.54
	Serious	0.21	0.30	0.50

Notes: The coefficients denote the change in log of effect size (pooled risk ratio (RR)) for the given category compared to the reference category. A negative value indicates that the log RR and RR are lower for studies belonging to the given category compared to the reference category.

(B) Evaluating the effect of specific sub-components of outcome and selection bias

Bias component	Domain	Coefficient	Standard error	p-value
Time Dependent Bias (yes, ref: no)	Outcome	-0.30	0.13	0.02
Immortal Time Bias (yes, ref: no)	Selection	0.24	0.17	0.15
Mortality Outcome Bias (yes, ref: no)	Outcome	-0.13	0.13	0.31
Mortality Exclusion Bias (yes, ref: no)	Selection	0.22	0.17	0.21

© 2025 Balasubramanian I et al. *JAMA Network Open*.

eReferences:

- 1. Zhang F, Chiu Y, Ensor J, Mohamed MO, Peat G, Mamas MA. Elixhauser outperformed Charlson comorbidity index in prognostic value after ACS: insights from a national registry. *J Clin Epidemiol*. Jan 2022;141:26-35. doi:10.1016/j.jclinepi.2021.08.025
- 2. Pope GC, Kautter J, Ellis RP, et al. Risk adjustment of Medicare capitation payments using the CMS-HCC model. *Health Care Financ Rev*. Summer 2004;25(4):119-41.
- 3. Escobar GJ, Greene JD, Scheirer P, Gardner MN, Draper D, Kipnis P. Risk-adjusting hospital inpatient mortality using automated inpatient, outpatient, and laboratory databases. *Med Care*. Mar 2008;46(3):232-9. doi:10.1097/MLR.0b013e3181589bb6
- 4. Choudhry SA, Li J, Davis D, Erdmann C, Sikka R, Sutariya B. A public-private partnership develops and externally validates a 30-day hospital readmission risk prediction model. *Online J Public Health Inform*. 2013;5(2):219. doi:10.5210/ojphi.v5i2.4726
- 5. Tung YC, Chang GM, Chang HY, Yu TH. Relationship between early physician follow-up and 30-day readmission after acute myocardial infarction and heart failure. Article. *PLoS ONE*. 2017;12(1)e0170061. doi:10.1371/journal.pone.0170061
- 6. Tak HJ, Chen LW, Wilson FA, et al. Post-Discharge Services for Different Diagnoses Than Index Hospitalization Predict Decreased 30-Day Readmissions Among Medicare Beneficiaries. Article. *Journal of General Internal Medicine*. 2019;34(9):1766-1774. doi:10.1007/s11606-019-05115-2
- 7. Anderson A, Mills CW, Willits J, et al. Follow-up Post-discharge and Readmission Disparities Among Medicare Fee-for-Service Beneficiaries, 2018. Article. *Journal of General Internal Medicine*. 2022;37(12):3020-3028. doi:10.1007/s11606-022-07488-3
- 8. Saxena FE, Bierman AS, Glazier RH, et al. Association of Early Physician Follow-up with Readmission among Patients Hospitalized for Acute Myocardial Infarction, Congestive Heart Failure, or Chronic Obstructive Pulmonary Disease. Article. *JAMA Network Open*. 2022:E2222056. doi:10.1001/jamanetworkopen.2022.22056
- 9. Hernandez AF, Greiner MA, Fonarow GC, et al. Relationship between early physician follow-up and 30-day readmission among medicare beneficiaries hospitalized for heart failure. Article. *JAMA*. 2010;303(17):1716-1722. doi:10.1001/jama.2010.533
- 10. Muus KJ, Knudson A, Klug MG, Gokun J, Sarrazin M, Kaboli P. Effect of post-discharge follow-up care on re-admissions among US veterans with congestive heart failure: a rural-urban comparison. Article. *Rural and remote health*. 2010;10(2):1447.
- 11. Tuso P, Watson HL, Garofalo-Wright L, et al. Complex case conferences associated with reduced hospital admissions for high-risk patients with multiple comorbidities. Article. *The Permanente journal*. 2014;18(1):38-42.
- 12. Lee KK, Jingrong Y, Hernandez AF, Steimle AE, Go AS, Yang J. Post-discharge Follow-up Characteristics Associated With 30-Day Readmission After Heart Failure Hospitalization. *Medical Care*. 2016;54(4):365-372. doi:10.1097/MLR.0000000000000492
- 13. McAlister FA, Youngson E, Kaul P, Ezekowitz JA. Early Follow-Up After a Heart Failure Exacerbation: The Importance of Continuity. *Circ Heart Fail*. Sep 2016;9(9)doi:10.1161/circheartfailure.116.003194
- 14. Murtaugh CM, Deb P, Zhu C, et al. Reducing Readmissions among Heart Failure Patients Discharged to Home Health Care: Effectiveness of Early and Intensive Nursing Services and Early Physician Follow-Up. Article. *Health Services Research*. 2017;52(4):1445-1472. doi:10.1111/1475-6773.12537
- 15. Huynh Q, Negishi K, De Pasquale C, et al. Effects of post-discharge management on rates of early re-admission and death after hospitalisation for heart failure. Article. *Medical Journal of Australia*. 2018;208(11):485-491. doi:10.5694/MJA17.00809
- 16. Bricard D, Or Z. Impact of early primary care follow-up after discharge on hospital readmissions. Article. *European Journal of Health Economics*. 2019;20(4):611-623. doi:10.1007/s10198-018-1022-y

© 2025 Balasubramanian I et al. *JAMA Network Open*.

- 17. Baecker A, Meyers M, Koyama S, et al. Evaluation of a transitional care program after hospitalization for heart failure in an integrated health care system. Article. *JAMA Network Open*. 2020;3(12)e20227410. doi:10.1001/jamanetworkopen.2020.27410
- 18. Distelhorst KS, Hansen DM. Neighborhood matters for transitional care and heart failure hospital readmission in older adults. *Geriatr Nurs*. Sep-Oct 2022;47:183-190. doi:10.1016/j.gerinurse.2022.07.014
- 19. Xu H, Granger BB, Drake CD, Peterson ED, Dupre ME. Effectiveness of Telemedicine Visits in Reducing 30-Day Readmissions Among Patients With Heart Failure During the COVID-19 Pandemic. Article. *Journal of the American Heart Association*. 2022;11(7)e023935. doi:10.1161/JAHA.121.023935
- 20. Balasubramanian I, Malhotra C. Can Timely Outpatient Visits Reduce Readmissions and Mortality Among Heart Failure Patients? Article. *Journal of General Internal Medicine*. 2024;doi:10.1007/s11606-024-08755-1
- 21. Sharma G, Kuo YF, Freeman JL, Zhang DD, Goodwin JS. Outpatient follow-up visit and 30-day emergency department visit and readmission in patients hospitalized for chronic obstructive pulmonary disease. Article. *Archives of Internal Medicine*. 2010;170(18):1664-1670. doi:10.1001/archinternmed.2010.345
- 22. Fidahussein SS, Croghan IT, Cha SS, Klocke DL. Posthospital follow-up visits and 30-day readmission rates in chronic obstructive pulmonary disease. Article. *Risk Management and Healthcare Policy*. 2014;7:105-112. doi:10.2147/RMHP.S62815
- 23. Sharif R, Parekh TM, Pierson KS, Kuo YF, Sharma G. Predictors of early readmission among patients 40 to 64 years of age hospitalized for chronic obstructive pulmonary disease. Article. *Annals of the American Thoracic Society*. 2014;11(5):685-694. doi:10.1513/AnnalsATS.201310-358OC
- 24. Hijjawi SB, Abu Minshar M, Sharma G. Chronic obstructive pulmonary disease exacerbation: A single-center perspective on hospital readmissions. *Postgraduate Medicine*. 2015;127(4):343-348. doi:10.1080/00325481.2015.1015394
- 25. Russo AN, Sathiyamoorthy G, Lau C, et al. Impact of a post-discharge integrated disease management program on COPD hospital readmissions. Article. *Respiratory Care*. 2017;62(11):1392-1402. doi:10.4187/respcare.05547
- 26. Budde J, Agarwal P, Mazumdar M, Braman SS. Follow-up soon after discharge may not reduce COPD readmissions. Article. *Chronic Obstructive Pulmonary Diseases*. 2019;6(2):129-131. doi:10.15326/jcopdf.6.2.2018.0149
- 27. Jiang L, Austin PC, Wodchis WP, Kiran T, Guan J, Gershon AS. Timing of follow-up visits after hospital discharge for COPD: Application of a new method. Article. *PLoS ONE*. 2024;19(7 July)e0302681. doi:10.1371/journal.pone.0302681
- 28. Hess CN, Shah BR, Peng SA, Thomas L, Roe MT, Peterson ED. Association of early physician follow-up and 30-day readmission after non-st-segment-elevation myocardial infarction among older patients. Article. *Circulation*. 2013;128(11):1206-1213. doi:10.1161/CIRCULATIONAHA.113.004569
- 29. Brown JR, Chang C-H, Zhou W, MacKenzie TA, Malenka DJ, Goodman DC. Health system characteristics and rates of readmission after acute myocardial infarction in the United States. *Journal of the American Heart Association*. 2014;3(3):e000714-e000714. doi:10.1161/JAHA.113.000714
- 30. Zabawa C, Cottenet J, Zeller M, et al. Thirty-day rehospitalizations among elderly patients with acute myocardial infarction Impact of postdischarge ambulatory care. Article. *Medicine (United States)*. 2018;97(24)e11085. doi:10.1097/MD.0000000000011085
- 31. Condon C, Lycan S, Duncan P, Bushnell C. Reducing Readmissions after Stroke with a Structured Nurse Practitioner/Registered Nurse Transitional Stroke Program. Article. *Stroke*. 2016;47(6):1599-1604. doi:10.1161/STROKEAHA.115.012524

© 2025 Balasubramanian I et al. *JAMA Network Open*.

- 32. Allen A, Barron T, Mo A, et al. Impact of Neurological Follow-Up on Early Hospital Readmission Rates for Acute Ischemic Stroke. *Neurohospitalist*. Jul 2017;7(3):127-131. doi:10.1177/1941874416684456
- 33. Terman SW, Reeves MJ, Skolarus LE, Burke JF. Association between early outpatient visits and readmissions after ischemic stroke. Article. *Circulation: Cardiovascular Quality and Outcomes*. 2018;11(4)e004024. doi:10.1161/CIRCOUTCOMES.117.004024
- 34. Leppert MH, Sillau S, Lindrooth RC, Poisson SN, Campbell JD, Simpson JR. Relationship between early follow-up and readmission within 30 and 90 days after ischemic stroke. Article. *Neurology*. 2020;94(12):e1249-e1258. doi:10.1212/WNL.0000000000009135
- 35. Hussein HM, Chrenka EA, Herrmann AA. Rate and Predictors of Acute Care Encounters in the First Month After Stroke. *Journal of Stroke and Cerebrovascular Diseases*. 2022/06/01/ 2022;31(6):106466. doi:https://doi.org/10.1016/j.jstrokecerebrovasdis.2022.106466
- 36. Bennett KJ, Probst JC, Vyavaharkar M, Glover SH. Lower Rehospitalization Rates Among Rural Medicare Beneficiaries With Diabetes. Article. *Journal of Rural Health*. 2012;28(3):227-234. doi:10.1111/j.1748-0361.2011.00399.x
- 37. Karunakaran A, Zhao H, Rubin DJ. Predischarge and Postdischarge Risk Factors for Hospital Readmission Among Patients With Diabetes. *Med Care*. Jul 2018;56(7):634-642. doi:10.1097/mlr.0000000000000931
- 38. Rubin DJ, Maliakkal N, Zhao H, Miller EE. Hospital Readmission Risk and Risk Factors of People with a Primary or Secondary Discharge Diagnosis of Diabetes. Article. *Journal of Clinical Medicine*. 2023;12(4)1274. doi:10.3390/jcm12041274
- 39. Johnson BH, Smoyer-Tomic KE, Siu K, et al. Readmission among hospitalized patients with nonvalvular atrial fibrillation. Article. *American Journal of Health-System Pharmacy*. 2013;70(5):414-422. doi:10.2146/ajhp120461
- 40. Hubbard M, Frost S, Siu K, Quon N, Esposito D. Association Between Outpatient Visits Following Hospital Discharge and Readmissions Among Medicare Beneficiaries With Atrial Fibrillation and Other Chronic Conditions. Article. *American Journal of Medical Quality*. 2014;29(3):206-212. doi:10.1177/1062860613493827
- 41. Brooke BS, Stone DH, Cronenwett JL, et al. Early primary care provider follow-up and readmission after high-risk surgery. Article. *JAMA Surgery*. 2014;149(8):821-828. doi:10.1001/jamasurg.2014.157
- 42. Saunders RS, Fernandes-Taylor S, Rathouz PJ, et al. Outpatient follow-up versus 30-day readmission among general and vascular surgery patients: a case for redesigning transitional care. *Surgery*. Oct 2014;156(4):949-56. doi:10.1016/j.surg.2014.06.041
- 43. Manji RA, Arora RC, Singal RK, Hiebert BM, Menkis AH. Early rehospitalization after prolonged intensive care unit stay post cardiac surgery: Outcomes and modifiable risk factors. Article. *Journal of the American Heart Association*. 2017;6(2)doi:10.1161/JAHA.116.004072
- 44. Poulose BK, Harris DA, Phillips S, et al. Reducing Early Readmissions after Ventral Hernia Repair with the Americas Hernia Society Quality Collaborative. Article. *Journal of the American College of Surgeons*. 2018;226(5):814-824. doi:10.1016/j.jamcollsurg.2018.01.048
- 45. Lumpkin ST, Strassle PD, Fine JP, Carey TS, Stitzenberg KB. Early Follow-up After Colorectal Surgery Reduces Postdischarge Emergency Department Visits. *Dis Colon Rectum*. Nov 2020;63(11):1550-1558. doi:10.1097/dcr.0000000000001732
- 46. Ghiam MK, Chyou DE, Dable CL, et al. 30-Day Readmissions and Coordination of Care Following Endoscopic Transsphenoidal Pituitary Surgery: Experience with 409 Patients. Review. *Journal of Neurological Surgery, Part B: Skull Base*. 2022;83:E410-E418. doi:10.1055/s-0041-1729980
- 47. Fair L, Squiers JJ, Misenhimer J, et al. In-Person Clinic Visits After Laparoscopic Cholecystectomy: Lessons Learned From COVID-19 Pandemic. Article. *Journal of Surgical Research*. 2023;291:396-402. doi:10.1016/j.jss.2023.06.029

© 2025 Balasubramanian I et al. *JAMA Network Open*.

- 48. Moneme AN, Wirtalla CJ, Roberts SE, Keele LJ, Kelz RR. Primary Care Physician Follow-Up and 30-Day Readmission after Emergency General Surgery Admissions. Article. *JAMA Surgery*. 2023;158(12):1293-1301. doi:10.1001/jamasurg.2023.4534
- 49. Kanwal F, Asch SM, Kramer JR, Cao Y, Asrani S, El-Serag HB. Early outpatient follow-up and 30-day outcomes in patients hospitalized with cirrhosis. Article. *Hepatology*. 2016;64(2):569-581. doi:10.1002/hep.28558
- 50. Schletzbaum M, Sweet N, Astor B, et al. Associations of Postdischarge Follow-Up With Acute Care and Mortality in Lupus: A Medicare Cohort Study. Article. *Arthritis Care and Research*. 2023;75(9):1886-1896. doi:10.1002/acr.25097
- 51. Leschke J, Panepinto JA, Nimmer M, Hoffmann RG, Yan K, Brousseau DC. Outpatient follow-up and rehospitalizations for sickle cell disease patients. Article. *Pediatric Blood and Cancer*. 2012;58(3):406-409. doi:10.1002/pbc.23140
- 52. Hazratjee N, Agito M, Lopez R, Lashner B, Rizk MK. Hospital readmissions in patients with inflammatory bowel disease. Article. *American Journal of Gastroenterology*. 2013;108(7):1024-1032. doi:10.1038/ajg.2012.343
- 53. Sbeit W, Khoury T, Kadah A, et al. Nonattendance to gastroenterologist follow-up after discharge is associated with a thirty-days re-admission in patients with inflammatory bowel disease: A multicenter study. Article. *Minerva Medica*. 2021;112(4):467-473. doi:10.23736/S0026-4806.21.07442-5
- 54. Berry SA, Fleishman JA, Yehia BR, et al. Thirty-day hospital readmission rate among adults living with HIV. Article. *AIDS*. 2013;27(13):2059-2068. doi:10.1097/QAD.0b013e3283623d5f
- 55. Hill L, Thompson C, Balcombe S, et al. Effects of a hospital discharge clinic among people with HIV: Lack of early follow-up is associated with 30-day hospital readmission and decreased retention in care. Article. *HIV Medicine*. 2024;25(3):332-342. doi:10.1111/hiv.13577
- 56. Saini E, Ali M, Du P, Crook T, Zurlo J. Early Infectious Disease Outpatient Follow-up of Outpatient Parenteral Antimicrobial Therapy Patients Reduces 30-Day Readmission. Article. *Clinical Infectious Diseases*. 2019;69(5):865-868. ciz073. doi:10.1093/cid/ciz073
- 57. Palms DL, Jacob JT. Close Patient Follow-up among Patients Receiving Outpatient Parenteral Antimicrobial Therapy. Article. *Clinical Infectious Diseases*. 2020;70(1):67-74. doi:10.1093/cid/ciz150
- 58. Dalton MK, Fox NM, Porter JM, Hazelton JP. Outpatient follow-up does not prevent emergency department utilization by trauma patients. Article. *Journal of Surgical Research*. 2017;218:92-98. doi:10.1016/j.jss.2017.05.076
- 59. Smith SM, Zhao X, Kenzik K, Michael C, Jenkins K, Sanchez SE. Scheduled Follow-Up and Association with Emergency Department Use and Readmission after Trauma. *J Am Coll Surg*. Sep 1 2024;239(3):234-241. doi:10.1097/xcs.0000000000001094
- 60. Deb P, Murtaugh CM, Bowles KH, et al. Does Early Follow-Up Improve the Outcomes of Sepsis Survivors Discharged to Home Health Care? Article. *Medical Care*. 2019;57(8):633-640. doi:10.1097/MLR.0000000000001152
- 61. Blank LJ, Van Hyfte G, Agarwal P, Mazumdar M, Jette N. Association of Outpatient Follow-Up With 30-Day Readmission After Epilepsy or Seizure Discharge in Medicare Beneficiaries Aged 65 and Older. *Neurology*. May 27 2025;104(10):e213638. doi:10.1212/wnl.0000000000213638
- 62. Misky GJ, Wald HL, Coleman EA. Post-hospitalization transitions: Examining the effects of timing of primary care provider follow-up. *J Hosp Med*. Sep 2010;5(7):392-7. doi:10.1002/jhm.666
- 63. Field TS, Ogarek J, Garber L, Reed G, Gurwitz JH. Association of Early Post-Discharge Follow-Up by a Primary Care Physician and 30-Day Rehospitalization Among Older Adults. Article in Press. *Journal of General Internal Medicine*. 2014;doi:10.1007/s11606-014-3106-4

• 64. Jackson C, Shahsahebi M, Wedlake T, DuBard CA. Timeliness of outpatient follow-up: an evidence-based approach for planning after hospital discharge. *Ann Fam Med*. Mar 2015;13(2):115-22. doi:10.1370/afm.1753
• 65. Bennett KJ, Probst JC. Thirty-Day Readmission Rates Among Dual-Eligible Beneficiaries. Article. *Journal of Rural Health*. 2016;32(2):188-195. doi:10.1111/jrh.12140
• 66. Wang H, Johnson C, Robinson RD, et al. Roles of disease severity and post-discharge outpatient visits as predictors of hospital readmissions. *BMC Health Services Research*. 2016;16:564-564. doi:10.1186/s12913-016-1814-7
• 67. Chakravarthy V, Ryan MJ, Jaffer A, et al. Efficacy of a Transition Clinic on Hospital Readmissions. Article. *American Journal of Medicine*. 2018;131(2):178-184.e1. doi:10.1016/j.amjmed.2017.08.037
• 68. Sinha S, Seirup J, Carmel A. Early primary care follow-up after ED and hospital discharge - does it affect readmissions? Article. *Hospital practice (1995)*. 2017;45(2):51-57. doi:10.1080/21548331.2017.1283935
• 69. Shen E, Koyama SY, Huynh DN, et al. Association of a Dedicated Post–Hospital Discharge Follow-up Visit and 30-Day Readmission Risk in a Medicare Advantage Population. *JAMA Internal Medicine*. 2017;177(1):132-135. doi:10.1001/jamainternmed.2016.7061
• 70. Toth M, Holmes M, Van Houtven C, Toles M, Weinberger M, Silberman P. Rural-Urban Differences in the Effect of Follow-Up Care on Postdischarge Outcomes. *Health Services Research*. 2017;52(4):1473-1493. doi:10.1111/1475-6773.12543
• 71. Ballard J, Rankin W, Roper KL, Weatherford S, Cardarelli R. Effect of Ambulatory Transitional Care Management on 30-Day Readmission Rates. Article. *American Journal of Medical Quality*. 2018;33(6):583-589. doi:10.1177/1062860618775528
• 72. Hawes EM, Smith JN, Pinelli NR, et al. Accountable Care in Transitions (ACTion): A Team-Based Approach to Reducing Hospital Utilization in a Patient-Centered Medical Home. Article. *Journal of Pharmacy Practice*. 2018;31(2):175-182. doi:10.1177/0897190017707118
• 73. Lam K, Abrams HB, Matelski J, Okrainec K. Factors associated with attendance at primary care appointments after discharge from hospital: a retrospective cohort study. Article. *CMAJ open*. 2018;6(4):E587-E593. doi:10.9778/cmajo.20180069
• 74. Tong L, Arnold T, Yang J, Tian X, Erdmann C, Esposito T. The association between outpatient follow-up visits and all-cause non-elective 30-day readmissions: A retrospective observational cohort study. Article. *PLoS ONE*. 2018;13(7)e0200691. doi:10.1371/journal.pone.0200691
• 75. Rayan-Gharra N, Shadmi E, Tadmor B, Flaks-Manov N, Balicer RD. Patients' ratings of the in-hospital discharge briefing and post-discharge primary care follow-up: The association with 30-day readmissions. Article. *Patient Education and Counseling*. 2019;102(8):1513-1519. doi:10.1016/j.pec.2019.03.018
• 76. Wiest D, Yang Q, Wilson C, Dravid N. Outcomes of a Citywide Campaign to Reduce Medicaid Hospital Readmissions With Connection to Primary Care Within 7 Days of Hospital Discharge. *JAMA Netw Open*. Jan 4 2019;2(1):e187369. doi:10.1001/jamanetworkopen.2018.7369
• 77. Baldino M, Bonaguro AM, Burgwardt S, et al. Impact of a Novel Post-Discharge Transitions of Care Clinic on Hospital Readmissions. *J Natl Med Assoc*. Apr 2021;113(2):133-141. doi:10.1016/j.jnma.2020.07.018
• 78. Nguyen HQ, Baecker A, Ho T, et al. Association between post-hospital clinic and telephone follow-up provider visits with 30-day readmission risk in an integrated health system. Article. *BMC Health Services Research*. 2021;21(1)826. doi:10.1186/s12913-021-06848-9
• 79. Patel SK, Miller A, Chen S, Lindsay A, Gray M, Su YP. Transitional Care Management Visits to Improve Coordination of Care. Article. *American Journal of Managed Care*. 2021;27(4):E130-E134. doi:10.37765/AJMC.2021.88622
• 80. Tak HJ, Goldsweig AM, Wilson FA, et al. Association of Post-discharge Service Types and Timing with 30-Day Readmissions, Length of Stay, and Costs. *JGIM: Journal of General Internal Medicine*. 2021;36(8):2197-2204. doi:10.1007/s11606-021-06708-6

- 81. Van De Graaf M, Patel H, Sheehan B, Ryal J. Implementation and Evaluation of a Team-Based Approach to Hospital Discharge Transition of Care. *PRiMER*. 2021;5:28. doi:10.22454/PRiMER.2021.675929
- 82. Kojima N, Bolano M, Sorensen A, et al. Cohort design to assess the association between post-hospital primary care physician follow-up visits and hospital readmissions. Article. *Medicine (United States)*. 2022;101(46):E31830. doi:10.1097/MD.0000000000031830
- 83. Schaub J, Shpilkerman YI, Roland H, Keyko K, Parisé K. Transitions of care for hospital discharges in a primary care network. Article. *Healthcare Management Forum*. 2022;35(3):147-152. doi:10.1177/08404704221084151
- 84. Boggs E, Misky G, Scarbro S, Gritz M, Tipirneni R, Lindrooth R. Disparities in postdischarge follow-up and risk of readmission between Medicaid and privately insured patients. Article. *Journal of Hospital Medicine*. 2024;doi:10.1002/jhm.13486
- 85. Brady N, Liang Y, Seidl KL, Marcozzi D, Stryckman B, Gingold DB. Association of Timely Outpatient Follow-Up and Readmission Risk in a Mobile Integrated Health Program. Article. *Population Health Management*. 2024;doi:10.1089/pop.2024.0020
- 86. Naseer M, Willers C, Boström AM, et al. Are Primary Health Care Visits Associated With Reduced Risk of Hospital Readmissions After Discharge From Geriatric Inpatient Departments? Evidence From Stockholm County. *J Prim Care Community Health*. Jan-Dec 2024;15:21501319241277413. doi:10.1177/21501319241277413
- 87. Sass J, Hampton D, Edward J, Cardarelli R. Evaluation of the Impact of Discharge Clinic Follow-Up Interventions on 30-Day Readmission Rates. *Popul Health Manag*. Apr 2024;27(2):137-142. doi:10.1089/pop.2023.0273
