# Supplementary material for: Outpatient Follow-Up and 30-Day Readmissions: A Systematic Review and Meta-Analysis
Source: JAMA Netw Open. 2025 Nov 4;8(11):e2541272. doi: 10.1001/jamanetworkopen.2025.41272 (PMC12587199; doi:10.1001/jamanetworkopen.2025.41272)
Supplement: Supplement 2. — Data Sharing Statement [file jamanetwopen-e2541272-s002.pdf]

Data Sharing Statement

Balasubramanian. Outpatient Follow-Up and 30-Day Readmissions. *JAMA Netw Open*.
Published November 04, 2025. doi:10.1001/jamanetworkopen.2025.41272

Data
Data available: Yes
Data types: Other (please specify)
Additional Information: The extracted data, data used for meta analysis
How to access data: Data can be accessed from the corresponding author chetna.malhotra@duke-nus.edu.sg
When available: With publication

Supporting Documents
Document types: None

Additional Information
Who can access the data: anyone requesting the data
Types of analyses: any purpose
Mechanisms of data availability: with investigator support
